# Receptor activity-modifying protein modulation of parathyroid hormone-1 receptor function and signaling

**DOI:** 10.3389/fphar.2024.1455231

**Published:** 2024-09-23

**Authors:** Paris Avgoustou, Ameera B. A. Jailani, Aditya J. Desai, David J. Roberts, Ewan R. Lilley, Grant W. Stothard, Timothy M. Skerry, Gareth O. Richards

**Affiliations:** Division of Clinical Medicine, University of Sheffield, Sheffield, United Kingdom

**Keywords:** parathyroid hormone receptor-1, parathyroid hormone, parathyroid hormone-related protein, receptor-activity-modifying protein, biased agonism

## Abstract

**Introduction:**

Receptor activity-modifying proteins (RAMPs) are known to modulate the pharmacology and function of several G-protein-coupled receptors (GPCRs), including the parathyroid hormone 1 receptor (PTH1R). However, the precise effects of different RAMPs on PTH1R signalling and trafficking remain poorly understood. This study investigated the impact of RAMP2 and RAMP3 on PTH1R function using a range of PTH and PTH-related protein (PTHrP)-derived ligands.

**Methods:**

We employed FRET imaging to assess PTH1R interactions with RAMPs. Cell surface expression of PTH1R was evaluated in the presence of RAMPs. PTH1R-mediated cAMP accumulation, β-arrestin recruitment, and calcium signalling were measured in response to various ligands. Antibody-capture scintillation proximity assays were used to examine G-protein activation patterns.

**Results:**

PTH1R preferentially interacted with RAMP2 and, to a lesser extent, RAMP3, but not with RAMP1. RAMP3 co-expression reduced cell surface expression of PTH1R. RAMP2 significantly enhanced PTH1R-mediated signalling responses to PTH (1-34), PTHrP (1-34), PTH (1-84), and PTH (1-17) analogue ZP2307, while RAMP3 co-expression attenuated or abolished these responses. Full-length PTHrP analogues exhibited lower potency and efficacy than PTHrP (1-34) in activating PTH1R. RAMP2 increased the potency and/or efficacy of these analogues, whereas RAMP3 reduced these responses. RAMP2 differentially modulated G-protein activation by PTH1R in a ligand-dependent manner, with PTH (1-34) and PTHrP (1-34) inducing distinct patterns of G-protein subtype activation.

**Discussion:**

These findings highlight the complex role of RAMPs in regulating PTH1R signalling and trafficking, revealing differential effects of RAMP2 and RAMP3 on receptor function. The data suggest that targeting the PTH1R/RAMP2 complex may be a promising strategy for developing novel bone anabolic therapies by leveraging biased agonism and functional selectivity. Further research using physiologically relevant models is needed to elucidate the therapeutic potential of this approach.

## Introduction

The G-protein-coupled receptors (GPCRs), a superfamily of seven transmembrane domain-containing receptors that consists of more than 800 members, are the largest family of membrane-bound proteins. GPCRs are involved in many physiological and pathophysiological processes (Hauser et al., 2017), and are among the most numerous targets for drug development (∼35% of all FDA-approved drugs, ∼700 drugs) (Hauser et al., 2017; Sriram and Insel, 2018).

**FIGURE 1:**
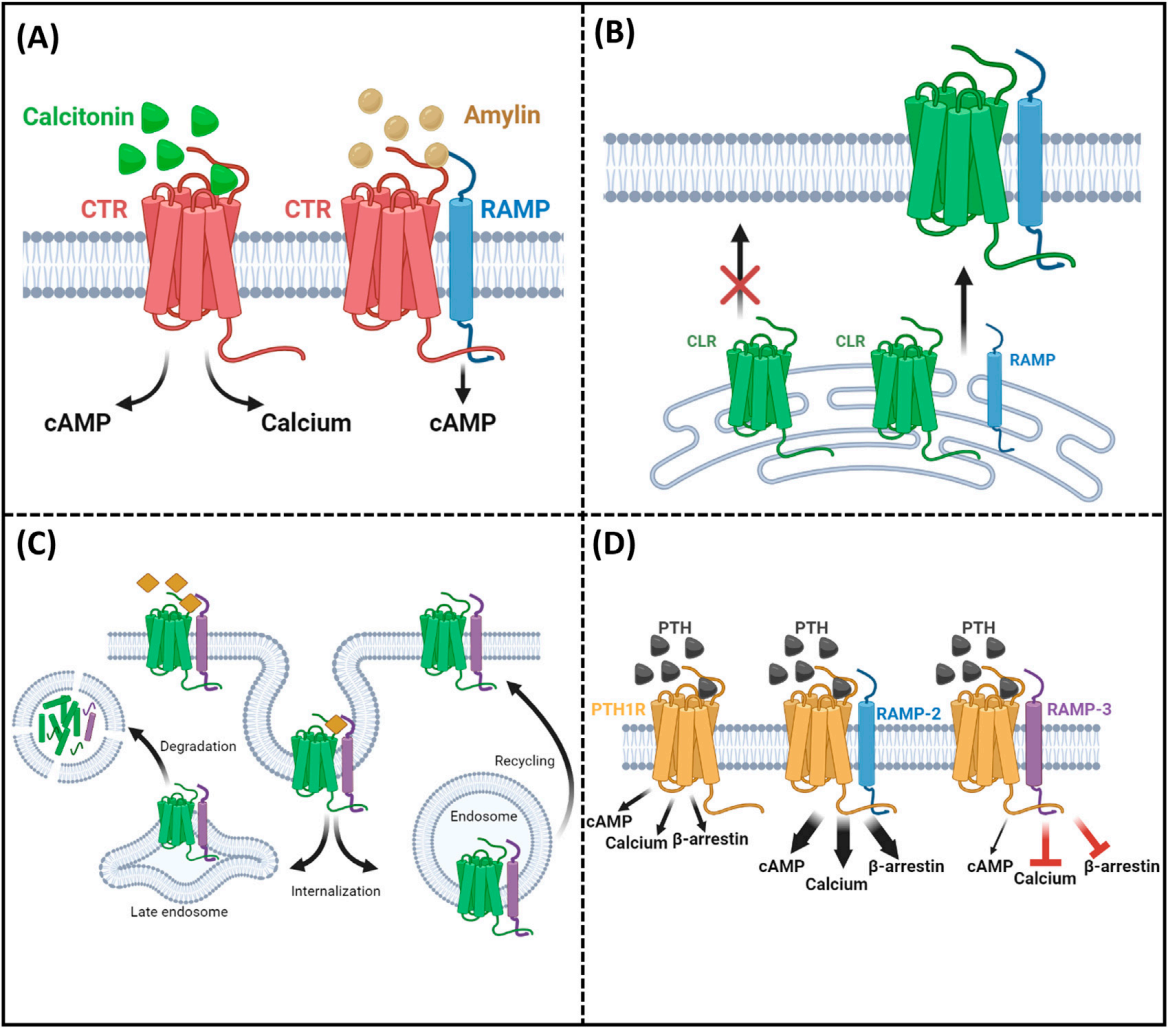
Summary of RAMP functions. **(A)** Ligand selectivity: RAMPs alter receptor–ligand binding preferences. **(B)** Receptor trafficking: RAMPs facilitate receptor localization to the cell surface. **(C)** Internalization and recycling: RAMPs influence receptor internalization and recycling rates. **(D)** Intracellular consequences/signaling: RAMPs modulate receptor-mediated signaling pathways.

The parathyroid hormone receptor-1 (PTH1R) is a member of this family and is more specifically a class-B GPCR, with a crucial role in a wide range of physiological systems including calcium homeostasis (in normal life, pregnancy, and lactation), skeletal development, and bone turnover. Pathological actions of PTH1R include involvement in the pathophysiology of osteoporosis, hypoparathyroidism (Zhao et al., 2019; Gardella and Vilardaga, 2015), humoral hypercalcemia of malignancy, and increasing numbers of malignancies (Guise and Mundy, 1996). These effects occur as a result of PTH1R’s ability to bind and transduce signals mediated by two cognate ligands. Parathyroid hormone (PTH) and PTH-related peptide (PTHrP) are the two endogenous agonists that lead to the activation of PTH1R and mediate its pleiotropic functions (Gardella and Vilardaga, 2015). Although PTH1R was classically thought to signal through the Gα_s_/cAMP pathway (activation) (Gardella and Vilardaga, 2015), it can also activate a number of other second-messenger cascades, including Gα_q_/calcium influx (Abousamra et al., 1992), Gα_i_/cAMP pathway (inhibition) (Miyauchi et al., 1990), and β-arrestins (Gesty-Palmer et al., 2006). Further complexity of this ligand/receptor system was added when PTH1R was found to interact with members of the receptor-activity-modifying protein (RAMP) family (Christopoulos et al., 2003; Lorenzen et al., 2019; Nemec et al., 2022). Mammalian RAMPs (RAMP1, RAMP2, and RAMP3) are single transmembrane domain proteins known to interact with several GPCR family members, altering and regulating receptor function and pharmacology (Weston et al., 2016; Hay et al., 2016; Hay and Pioszak, 2016) but with no ligand-binding activities alone, as illustrated in Figure 1. RAMPs regulate ligand selectivity for a small number of GPCRs (Figure 1A) (Poyner et al., 2002). This discovery stemmed from their capacity to alter the ligand selectivity of the calcitonin-like receptor and calcitonin receptor. The most well-characterized example of this is how RAMP expression can transform a calcitonin receptor’s phenotype, causing it to bind with amylin instead of calcitonin. This shift in binding preference leads to the activation of different intracellular signaling pathways.

Beyond ligand selectivity, RAMPs are involved in the intracellular trafficking of rather more receptors, albeit putatively (Figure 1B) (McLatchie et al., 1998). For some receptors, RAMPs are absolutely essential for proper localization at the cell surface. A prime example is the calcitonin receptor-like receptor (CLR), which can only function when it is trafficked in association with one of the three types of RAMPs. Moreover, it was shown that RAMPs are involved in the regulation of GPCR internalization and recycling (Figure 1C) (Bomberger et al., 2005; Mackie et al., 2019). Although some receptors can traffic without RAMP association, RAMPs can influence the rate and kinetics of this transport. Recent studies have shed light on how RAMPs affect GPCR recycling rates. For instance, RAMP3 has been implicated in the rapid recycling of the atypical chemokine receptor ACKR3, demonstrating their importance in regulating receptor presence at the cell surface (Mackie et al., 2019). In addition, it has been shown that RAMPs can modulate receptor downstream signaling when responding to the same ligands (Pioszak and Hay, 2020) (Figure 1D). This means that RAMPs can alter the intracellular consequences of a ligand binding to its receptor. A striking example of this is observed with the PTH1R receptor. When RAMP2 is present, there are significant increases in second messenger activation, including cAMP, calcium, and β-arrestin, compared to when PTH1R is alone, as shown by Nemec et al. (2022).

Most of the studies to date have focused on the interaction between PTH1R and RAMP2 (Christopoulos et al., 2003; Lorenzen et al., 2019); however, some studies include data on the interactions of PTH1R with RAMP3 (Nemec et al., 2022; Harris et al., 2021). Nemec et al. (2022) have shown that RAMP2 alters the PTH1R signaling in an agonist-dependent manner, with the most significant increase in the PTH-mediated Gα_i3_ signaling sensitivity. Furthermore, RAMP2 caused an increase in both PTH (1-34)- and PTHrP (1-34)-triggered β-arrestin recruitment to PTH1R.

The physiological consequences of PTH1R/RAMP interaction are still unclear. Evidence from RAMP2 knockout mice (RAMP2^+/−^) showed a decrease in PTH1R expression as well as a dampened response on serum phosphate concentration after systemic parathyroid hormone (PTH) administration; however, it is worth mentioning that in this study, a very large dose of PTH (500 μg/kg) was used (Kadmiel et al., 2017). In the same study, placental dysfunction and defects in arterial remodeling were observed in RAMP2+/− mice that were not associated with the RAMP2/CLR receptor complex, suggesting a possible physiological role of the PTH1R/RAMP2 receptor complex (Kadmiel et al., 2017).

In this study, we have confirmed and added to the observations by Nemec et al. and expanded on the repertoire of PTH-derived ligands and the signaling consequences of both PTH1R/RAMP2 and PTH1R/RAMP3 interactions (Figure 1D).

## Results

### PTH1R and RAMPs interactions and cell surface trafficking

To identify which RAMPs have potential for functional interactions with PTH1R, we used a sensitized fluorescent resonance energy transfer (FRET) technique to determine the receptor/RAMP interactions closer than 10 nm at the cell surface in COS-7 cells transfected with different RAMPs and receptors. We also used FRET-based stoichiometric analysis (Hoppe et al., 2002) to determine the fraction of receptor and RAMPs in the FRET complex.

**FIGURE 2:**
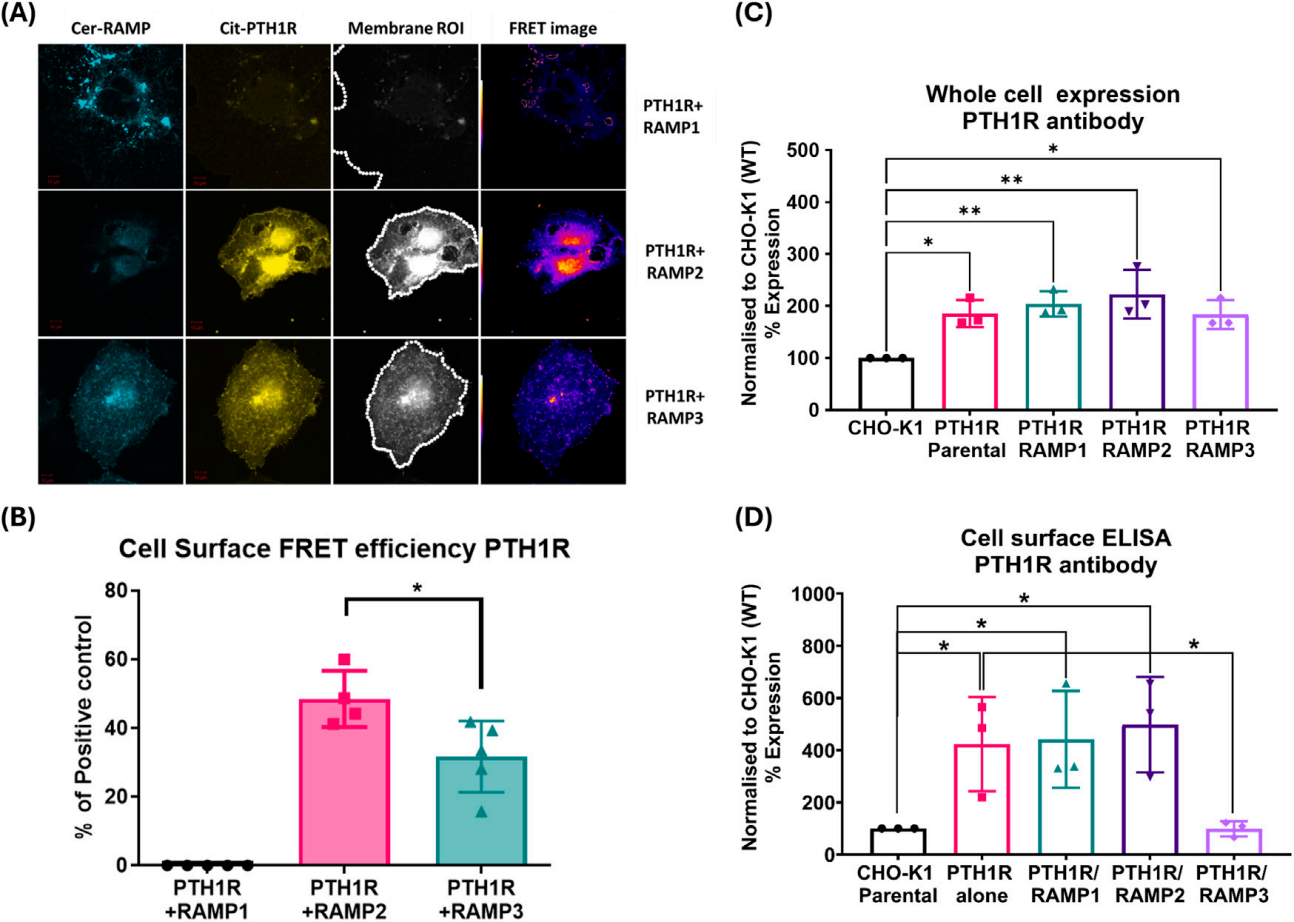
PTH1R and RAMPs interaction. Membrane localization of PTH1R and RAMPs using FRET imaging of COS-7 cells transfected with Cerulean-RAMPs 1-3 and Citrine-PTH1R combinations. Analysis of cell surface FRET was performed using a series of 50-pixel regions of interest (ROI) constructed around the Citrine-receptor raw image to cover the entire cell membrane. Images are representative of 4–6 replicate measurements. **(A)** Confocal images of RAMP1-3 (Cerulean) PTH1R (Citrine) in COS-7 cell. **(B)** FRET efficiencies of RAMP1-3 with PTH1R represented as % of the maximum FRET calculated using the Cerulean–Citrine fusion construct. The data represent mean ± SEM, n = 4; *p < 0.05 and **p < 0.001, Mann–Whitney test. Cell-based ELISA against PTH1R in the presence and absence of RAMPs. **(C)** Total PTH1R expression was determined in CHO-K1 cells (permeabilized) stably expressing PTH1R alone (mock), PTH1R/RAMP1, PTH1R/RAMP2, PTH1R/RAMP3, and the parental cell line. **(D)** Cell surface ELISA was performed on cells stably expressing PTH1R alone (mock), PTH1R/RAMP1, PTH1R/RAMP2, PTH1R/RAMP3, and the parental cell line (CHO-K1) against PTH1R antibody. Expression was normalized to CHO-K1 parental cell line. Data are derived from three replicate measurements in three independently replicated studies. Data are presented as mean ± SD. Comparisons were analyzed using unpaired Student’s t-test, *p < 0.05.

FRET was quantified at the cell surface using membrane ROIs, as shown in Figure 2A. This showed that PTH1R interacts with RAMP2 and to a lesser extent with RAMP3; however, no interaction was observed with RAMP1 (Figures 2A, B). The relative stoichiometry between PTH1R/RAMP2 and RAMP3 also differed (Table 1), suggesting that PTH1R and RAMP2 formed ∼1:1 complex; however, PTH1R and RAMP3 formed ∼1:2 complex (Table 1).

**TABLE 1:** Mean values for NFRET and the FRET stoichiometric constants for various RAMP and receptor combinations on the cell surface.

	Cell membrane NFRET relative to cer-cit fusion in %	Fa (%)	Fd (%)	R
PTH1R/RAMP1	NA	NA	NA	NA
PTH1R/RAMP2	48.8 ± 4.1*	52.2 ± 10.5	55.3 ± 5.0*	1.27 ± 0.31
PTH1R/RAMP3	32.0 ± 4.7	67.0 ± 4.7	24.8 ± 4.3	0.37 ± 0.06

Fa, fraction of GPCR (acceptor) in FRET complex; Fd, fraction of RAMP (donor) in FRET complex; R, molar ratio of acceptor to donor; NA indicates no detectable FRET.

To support if RAMP interactions alter cell surface trafficking of PTH1R, we performed cell-based ELISA for PTH1R in the presence and absence of RAMPs (Bailey and Hay, 2007). These studies were performed in stably expressing cells, as described in the methods section (see Supplementary Methods and Supplementary Figures S1, S2 for stable cell line generation and characterization for RAMP expression). The expression of total PTH1R was not altered by the presence of RAMP1, RAMP2, or RAMP3 (Figure 2C). The presence of RAMP1 or RAMP2 did not alter the cell surface translocation of PTH1R, compared to PTH1R parental cells (Figure 2D). However, when co-expressed with RAMP3, cell surface levels of PTH1R were comparable to the parental cell line (CHO-K1), suggesting that RAMP3 may play a role in intracellular retention of PTH1R (Figure 2D).

### Consequences of RAMP2 interaction on PTH1R G-protein response to ligand activation

To explore the consequences of PTH1R/RAMP2 interaction, we used antibody-capture scintillation proximity assays (SPA) to measure the spectra of activation of individual G-proteins (Gα_i_, Gα_q_, and Gα_s_) by PTH1R alone or in combination with RAMP2 (Figure 2). These studies were controlled for the levels of receptor number using radioligand binding studies (Supplementary Methods, Supplementary Table S1), showing no difference in pKd and Bmax between PTH1R alone and PTH1R/RAMP2.

**FIGURE 3:**
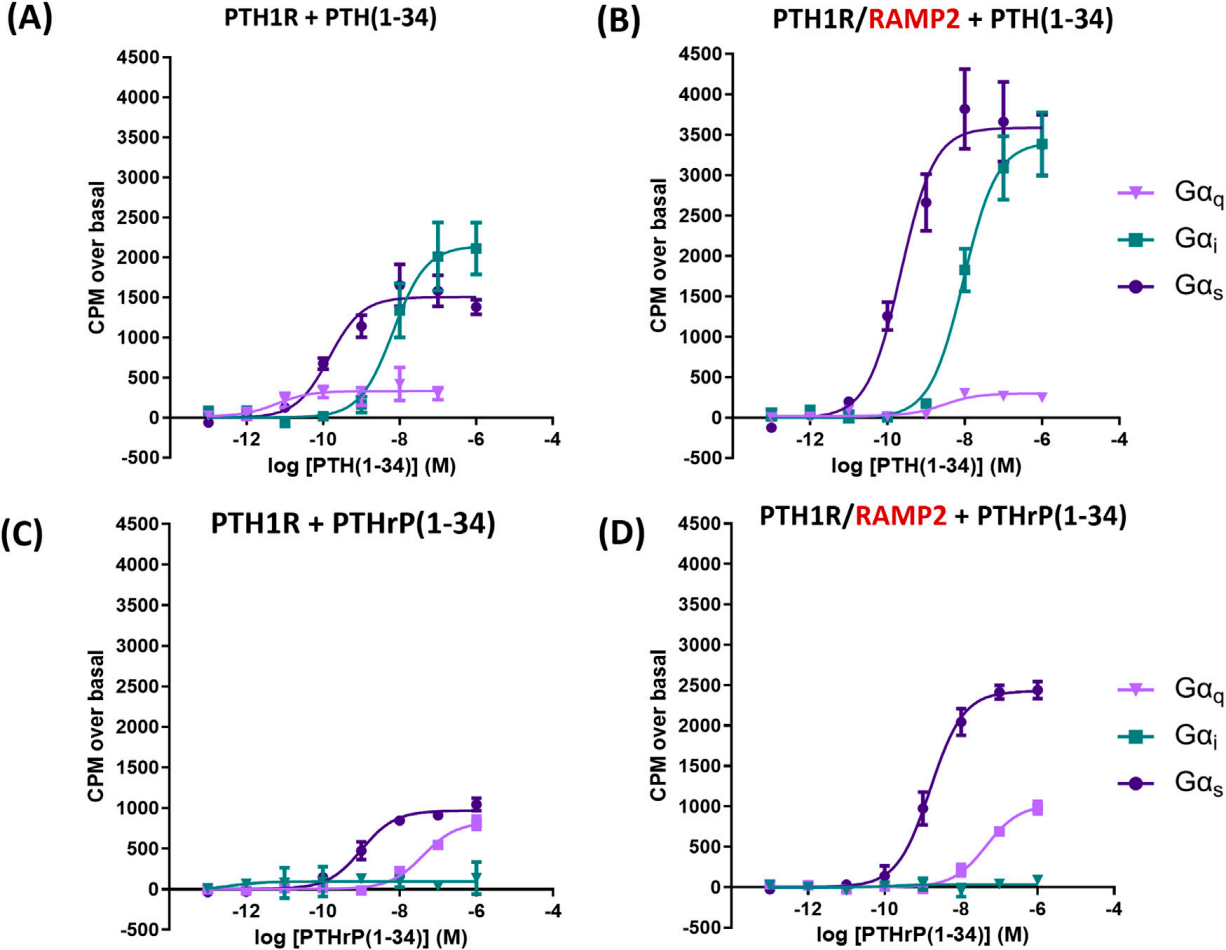
Antibody-capture scintillation proximity assays (SPA). Dose–response curves constructed from experiments in which different concentrations of ligand [PTH (1-34) or PTHrP (1-34)] were incubated with 10 µg of membrane preparations from COS-7 cells transfected either with PTH1R alone or PTH1R and RAMP2. Disclosure of G-protein activation was performed by the addition of [^35^S]GTPɣ^S^ and scintillation beads to allow measurement. **(A)** Responses of membranes from cells transfected with PTH1R to PTH (1-34). **(B)** Responses of membranes from cells transfected with PTH1R/RAMP2 to PTH (1–34). **(C)** Responses of the same membranes as a, transfected with PTH1R, to PTHrP (1-34). **(D)** Responses of the same membranes as b, transfected PTH1R/RAMP2, to PTHrP (1-34). Data are derived from curves constructed from three independently replicated studies, each consisting of two replicate measurements at each of the eight ligand concentrations. Table data shown as -log values ±SEM with nM values in brackets. Comparisons were analyzed using ANOVA with Bonferroni *post hoc* analysis (*p < 0.05, **p < 0.01, and ***p < 0.001).

In COS-7 cells’ membrane preparations expressing PTH1R alone, we assessed the effects of varying concentrations of PTH (1-34) and PTHrP (1-34) in the absence of RAMPs. PTH (1-34) induced a greater maximal activation (efficacy) of Gα_s_ (39%) and Gα_i_ (67%) than PTHrP (1-34) and a detectable activation in Gα_q_ that is absent with PTHrP (all *p* < 0.05) (Figures 3A, C; Table 2).

**TABLE 2:** Potency (pEC50) and efficacy of G-protein activation derived from the curves obtained in SPA studies (Figure 3).

	PTH(1-34)			
	PTH1R		PTH1R/RAMP2	
	pEC50 ± SEM.	Efficacy (CPM) ± SEM.	pEC50 ± SEM.	Efficacy (CPM) ± SEM.
Gαs	9.82 ± 0.09 (0.15)	1,582 ± 149	9.64 ± 0.07 (0.23)	3,765 ± 411**
Gαi	8.09 ± 0.14 (8.13)	2,225 ± 302	8.02 ± 0.05 (9.50)	3,536 ± 290**
Gαq	11.47 ± 0.19 (0.003)	281 ± 27	8.82 ± 0.09 (1.52)**	219 ± 31

	PTHrP(1-34)			
	PTH1R		PTH1R/RAMP2	
	pEC50 ± SEM.	Efficacy (CPM) ± SEM.	pEC50 ± SEM.	Efficacy (CPM) ± SEM.
Gαs	8.99 ± 0.17 (1.03)	970 ± 43	8.79 ± 0.16 (1.63)	2,441 ± 87**
Gαi	NA	NA	NA	NA
Gαq	7.41 ± 0.12 (39.20)	821 ± 66	7.36 ± 0.05 (43.45)	1,016 ± 72

Data are derived from curves constructed from three independently replicated studies, each consisting of two replicate measurements at each of the eight ligand concentrations. Data are shown as -log values ±SEM with nM values in brackets.

Stimulation of [35 S] GTPƴS binding to G-protein subtypes by PTH (1-34) and PTHrP (1-34) at PTHR1 receptor in the presence and absence of RAMP2; NA: not applicable.

We then assessed the effects of RAMP2 on PTH1R G-protein response to PTH (1-34) and PTHrP (1-34) activation. The interaction of PTH1R with RAMP2 increased PTH (1-34)-stimulated maximal activation (efficacy) of Gα_s_ (by 140%) and Gα_i_ (by 60%), without changing Gα_q_ (Figures 3A, B; Table 2), compared with the same ligand acting on the receptor alone. There were no changes in potency (EC_50_) for Gα_s_ and Gα_i_ activation, but PTH-stimulated Gα_q_ potency was significantly reduced in PTH1R associated with RAMP2 compared with that in PTH1R alone. In contrast, PTHrP (1-34) induced a different pattern of changes in the same membrane preparations expressing PTH1R and RAMP2 compared to PTH1R alone. PTHrP-stimulated Gα_s_ efficacy was increased by ∼150% without changes in Gα_i_ or Gα_q_ or any changes in potency (Figures 3C, D; Table 2).

### RAMP2 modulates receptor functionality in a ligand-dependent manner

Following on from the interaction and G-protein activation studies, we decided to investigate the effects of RAMP2 and RAMP3 on PTH1R second messenger activation in response to PTH (1-84), PTH (1-34), PTHrP (1-34), PTHrP (1-108), and PTHrP (1-141), and cyclic PTH (1-17), to expand on the repertoire of PTH-derived ligands (Supplementary Figure S3).

### PTH (1-34)

**FIGURE 4:**
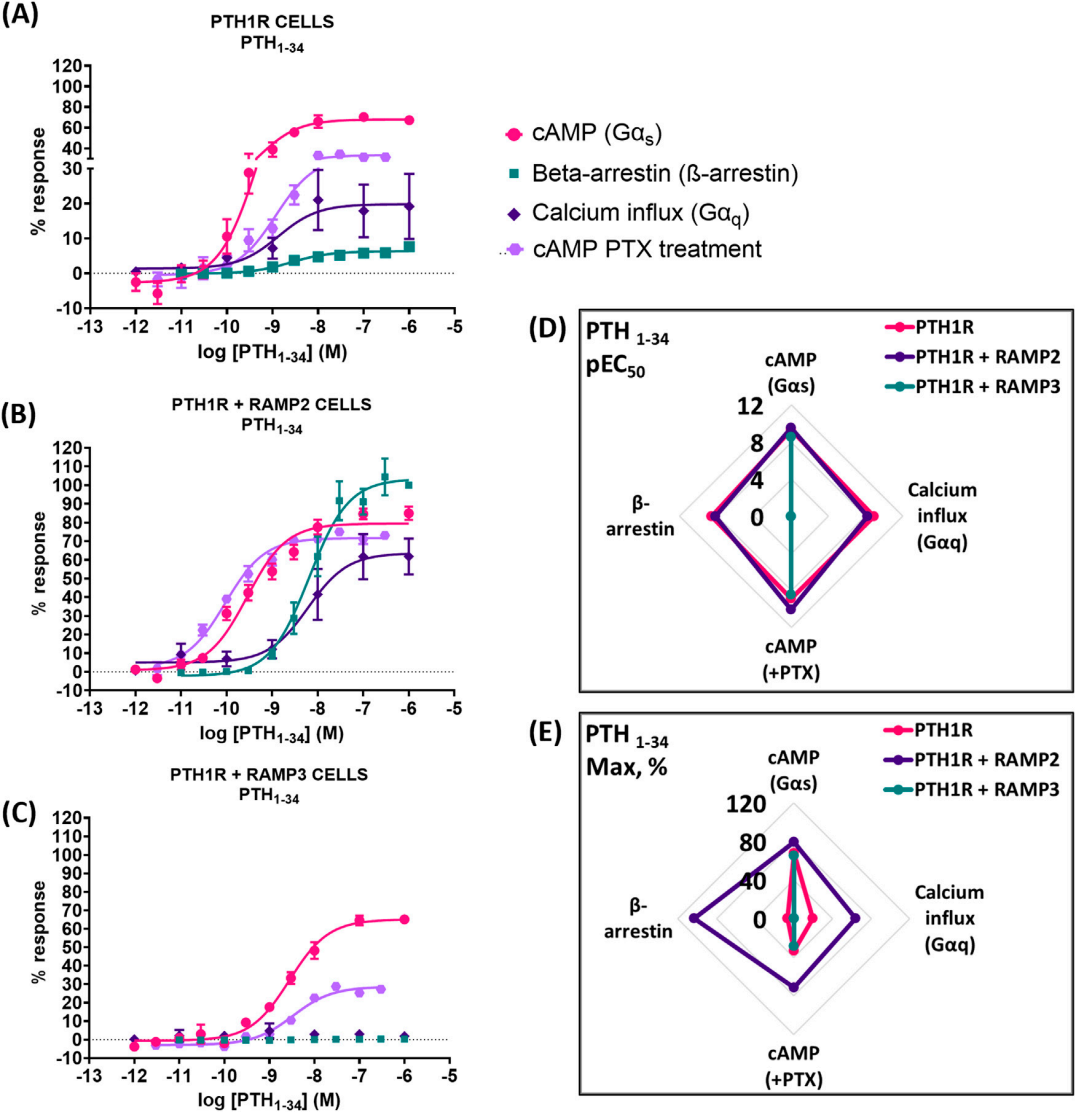
Consequences of RAMP2 and RAMP3 in the potency and efficacy of PTH (1-34) in different functional assays. Dose–response curves of PTH (1-34) in different second messenger pathways: in CHO-K1 cells overexpressing **(A)** PTH1R alone (mock), **(B)** PTH1R with RAMP2, and **(C)** PTH1R with RAMP3. Spider diagrams of **(D)** the potency and **(E)** the efficacy of PTH (1–34) in those assays, as extracted from the dose–response curves. Data are derived from curves constructed from at least 3–4 independently replicated studies, each consisting of two replicate measurements at each of the 11 ligand concentrations. Data were analyzed using comparison of fits (GraphPad Prism) for non-linear regression curves and three-parameter logistic curve (*p < 0.05, **p < 0.01 ***p < 0.001). All curves were expressed as a % of the positive controls/maximal response. Controls: cAMP studies: forskolin (100 μM), calcium influx studies: ATP (100 μM), β-arrestin-2 recruitment studies: maximal response at highest dose (1 μM).

PTH1R/RAMP2 cells showed a significant increase in both potency and maximal response (efficacy) to PTH (1-34)-mediated cyclic AMP (cAMP) accumulation, compared to PTH1R alone (Figure 4; Table 3). In contrast, PTH1R/RAMP3 cells showed a significant reduction in potency but no changes in efficacy compared to PTH1R alone (Figure 4; Table 3). Even though there was no significant difference in the potency of PTH (1-34) to recruit β-arrestin between PTH1R alone and PTH1R/RAMP2 cells, there was a significant increase in the efficacy of the ligand [PTH (1-34)] in the RAMP2-transfected cells (Figure 4; Table 3). RAMP3-transfected cells had no detectable response to PTH (1-34) in the β-arrestin assays. Similarly, when the same cells were used to measure calcium influx changes, a significant difference in efficacy was shown between PTH1R alone and PTH1R/RAMP2 cells, but there was no significant change in potency (Figure 4; Table 3). RAMP3-transfected cells had no response to PTH (1-34) in all the assays we performed. To assess the effects of RAMP2 in Gα_i_ response, the cells were treated with PTX (pertussis toxin) prior to performing cAMP accumulation assays. In these assays, the RAMP2-transfected cells showed a significant increase in both the potency and efficacy of PTH (1-34), compared to PTH1R cells alone. RAMP3 had no significant effect (Figure 4; Table 3).

**TABLE 3:** Consequences of RAMP2 and RAMP3 in the potency and efficacy of PTH (1-34) in different functional assays (Figure 4).

Cell line	pEC50 ± SEM			
	cAMP (Gαs)	ß-arrestin	Calcium influx (Gαq)	cAMP + PTX
PTH1R parental	9.30 ± 0.10	8.60 ± 0.19	8.90 ± 0.66	8.90 ± 0.12
PTH1R + RAMP2	9.55 ± 0.07***	8.19 ± 0.09	8.21 ± 0.29	10.03 ± 0.07***
PTH1R + RAMP3	8.55 ± 0.07***	ND	ND	8.48 ± 0.10

Cell line	Efficacy (%) ± SEM			
	cAMP (Gαs)	ß-arrestin	Calcium influx (Gαq)	cAMP + PTX
PTH1R parental	68.1 ± 2.5	6.31 ± 0.42	19.8 ± 3.80	33.5 ± 1.50
PTH1R + RAMP2	79.4 ± 1.91***	103.4 ± 3.74***	63.5 ± 6.35**	71.6 ± 1.20**
PTH1R + RAMP3	65.1 ± 2.05	ND	ND	28.7 ± 1.37

ND: could not be determined.

Data were analyzed using comparison of fits (GraphPad Prism) for non-linear regression curves and three-parameter logistic curve (*P < 0.05, **P < 0.01, and ***P < 0.001).

### PTHrP (1-34)

**FIGURE 5:**
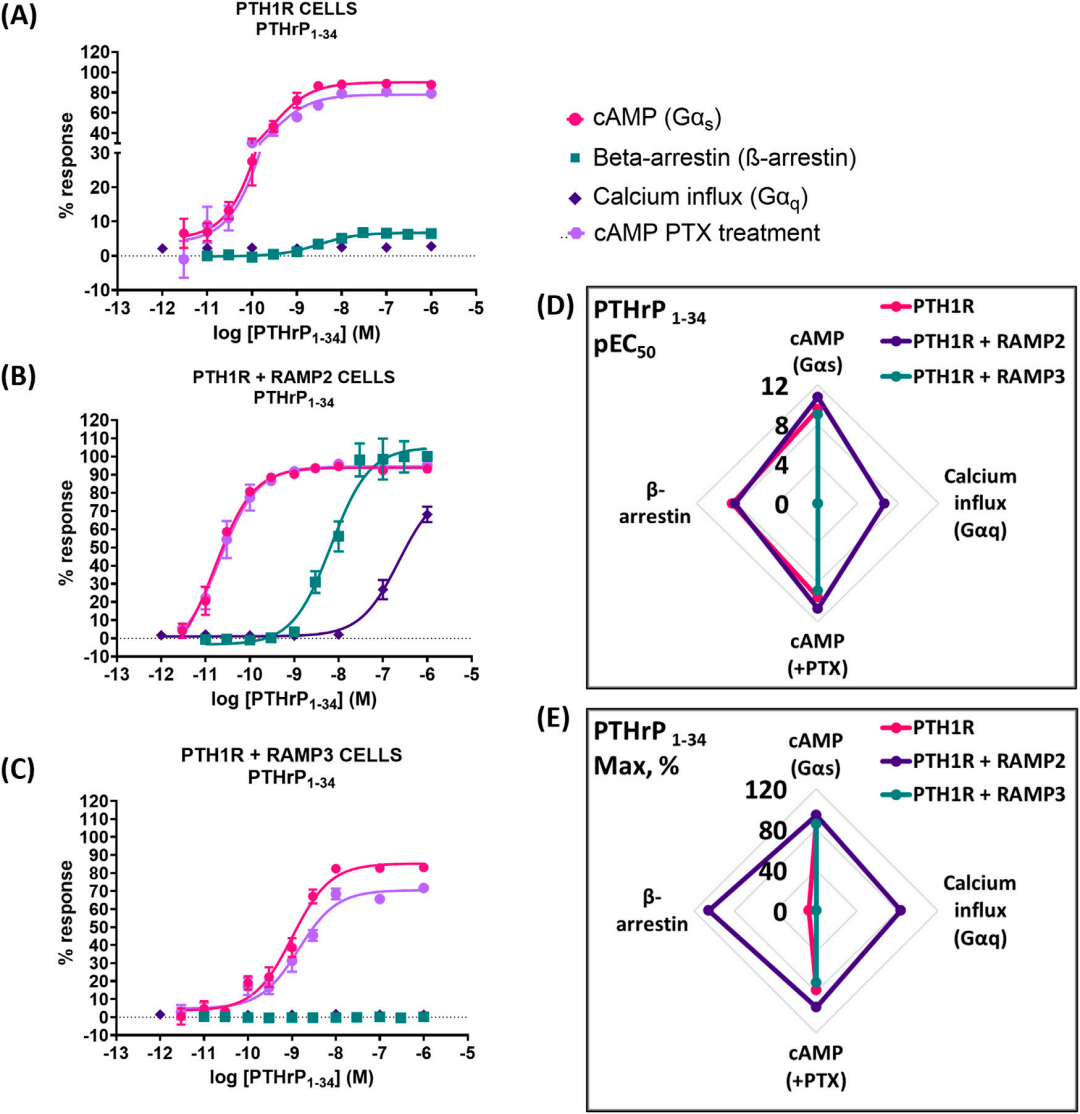
Consequences of RAMP2 and RAMP3 in the potency and efficacy of PTHrP (1-34) in different functional assays. Dose–response curves of PTHrP (1-34) in different second messenger pathways: in CHO-K1 cells overexpressing **(A)** PTH1R alone (mock), **(B)** PTH1R with RAMP2, and **(C)** PTH1R with RAMP3. Spider diagrams of **(D)** the potency and **(E)** the efficacy of PTHrP (1–34) in those assays, as extracted from the dose–response curves. Data are derived from curves constructed from at least 3–4 independently replicated studies, each consisting of two replicate measurements at each of the 11 ligand concentrations. Data were analyzed using comparison of fits (GraphPad Prism) for non-linear regression curves and three-parameter logistic curve (*p < 0.05, **p < 0.01 ***p < 0.001). All curves were expressed as a % of the positive controls/maximal response. Controls: cAMP studies: forskolin (100 μM), calcium influx studies: ATP (100 μM), β-arrestin-2 recruitment studies: maximal response at highest dose (1 μM).

When PTH1R RAMP2 cells were stimulated with PTHrP (1-34), a significant increase in potency but not efficacy was shown in cAMP accumulation, compared to PTH1R alone (Figure 5; Table 4). On the other hand, transfection with RAMP3 showed a significant reduction in potency, but there were no changes in efficacy compared to PTH1R alone (Figure 5; Table 4). Similarly, with the mediated effects of PTH (1-34) on β-arrestin, a significant increase in the efficacy of the ligand [PTHrP (1-34)] in the RAMP2-transfected cells was shown (Figure 5; Table 4). In addition, RAMP3-transfected cells had no response to PTHrP (1-34) in the β-arrestin assays (Figure 5; Table 4). When stimulated with PTHrP (1-34), only cells transfected with RAMP2 showed an increase in calcium influx, whereas PTH1R alone and PTH1R RAMP3 cells showed no response (Figure 5; Table 4). In Gα_i_ response, RAMP2 caused a significant increase in both potency and efficacy of PTHrP (1-34) compared to PTH1R alone. On the other hand, RAMP3 caused a significant reduction in both potency and efficacy (Figure 5; Table 4).

**TABLE 4:** Consequences of RAMP2 and RAMP3 in the potency and efficacy of PTHrP (1-34) in different functional assays (Figure 5).

Cell line	pEC50 ± SEM			
	cAMP (Gαs)	ß-arrestin	Calcium influx (Gαq)	cAMP + PTX
PTH1R parental	9.55 ± 0.08	8.50 ± 0.16	ND	9.57 ± 0.09
PTH1R + RAMP2	10.8 ± 0.07***	8.17 ± 0.09	6.65 ± 0.09*	10.7 ± 0.10***
PTH1R + RAMP3	9.00 ± 0.08***	ND	ND	8.85 ± 0.09***

Cell line	Efficacy (%) ± SEM			
	cAMP (Gαs)	ß-arrestin	Calcium influx (Gαq)	cAMP + PTX
PTH1R parental	90.1 ± 2.26	6.75 ± 0.40	ND	78.0 ± 2.30
PTH1R + RAMP2	93.9 ± 1.31	105.1 ± 3.69***	83.4 ± 5.00*	94.5 ± 1.91***
PTH1R + RAMP3	85.3 ± 2.45	ND	ND	70.5 ± 2.55*

ND: could not be determined.

Data were analyzed using comparison of fits (GraphPad Prism) for non-linear regression curves and three-parameter logistic curve (*P < 0.05, **P < 0.01, and ***P < 0.001).

### PTH (1-84)

**FIGURE 6:**
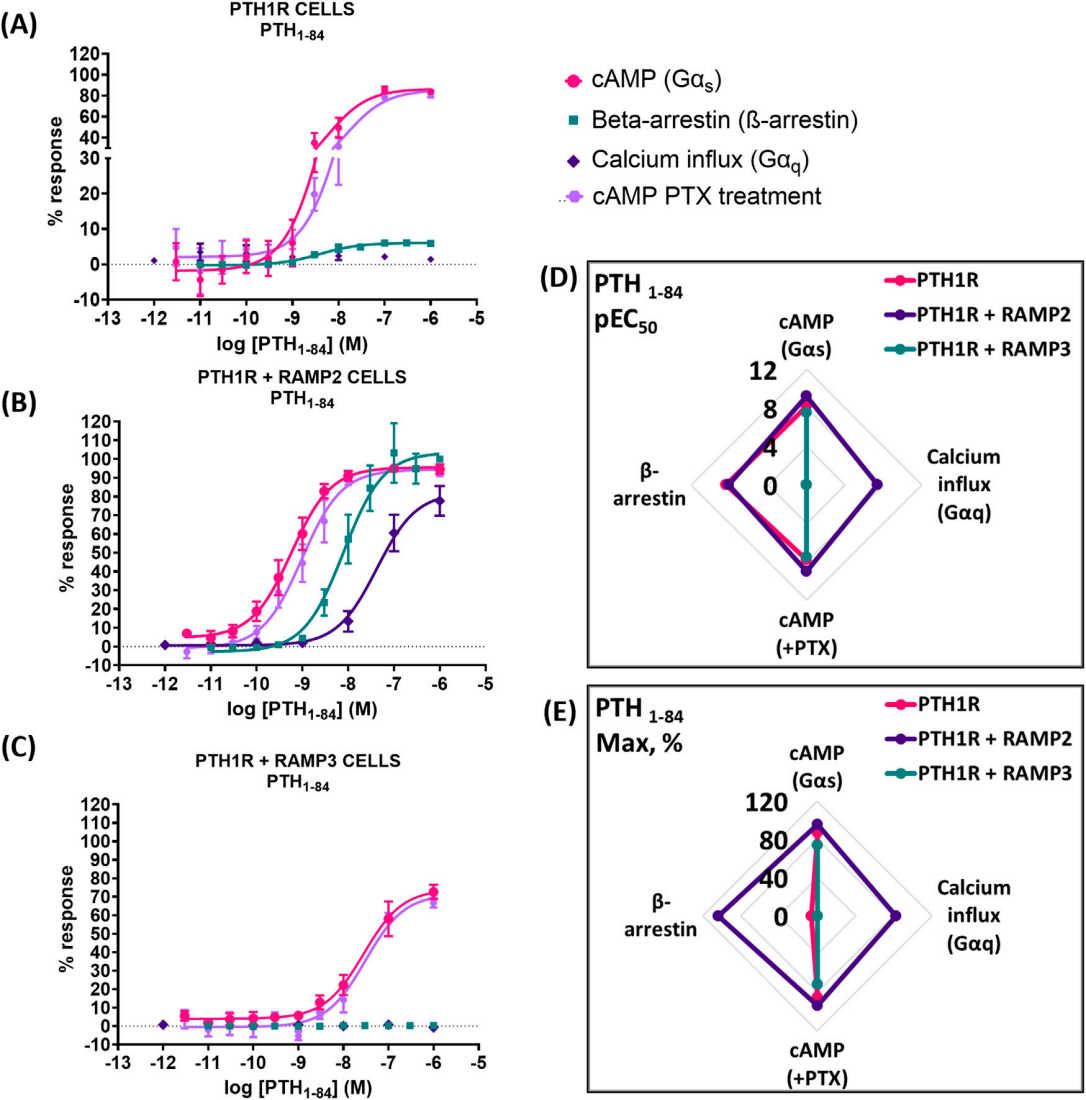
Consequences of RAMP2 and RAMP3 in the potency and efficacy of PTH (1-84) in different functional assays. Dose–response curves of PTH (1-84) in different second messenger pathways: in CHO-K1 cells overexpressing **(A)** PTH1R alone (mock), **(B)** PTH1R with RAMP2, and **(C)** PTH1R with RAMP3. Spider diagrams of **(D)** the potency and **(E)** the efficacy of PTH (1-84) in those assays as extracted from the dose–response curves. Data are derived from curves constructed from at least 3–4 independently replicated studies, each consisting of two replicate measurements at each of the 11 ligand concentrations. Data were analyzed using comparison of fits (GraphPad Prism) for non-linear regression curves and three-parameter logistic curve (*p < 0.05, **p < 0.01 ***p < 0.001). All curves were expressed as a % of the positive controls/maximal response. Controls: cAMP studies: forskolin (100 μM), calcium influx studies: ATP (100 μM), β-arrestin-2 recruitment studies: maximal response at highest dose (1 μM).

While using the intact biologically active 84 amino acid peptide (PTH (1-84)), similar effects were observed. RAMP2-transfected cells (PTH1R RAMP2) showed a significant increase in potency in cAMP accumulation (Gα_s_) and in Gα_i_ responses, but they showed no changes in efficacy (Figure 8). On the other hand, a significant increase in efficacy of PTH (1-84) was shown in β-arrestin. However, there were no changes in potency (Figure 6; Table 5). Similarly, with PTHrP (1-34), only cells transfected with RAMP2 showed an increase in calcium influx, whereas PTH1R alone and PTH1R RAMP3 cells showed no response when stimulated with PTH (1-84) (Figure 6; Table 5). Transfection with RAMP3 resulted in a significant decrease in both potency and efficacy PTH (1-84) in cAMP accumulation (Gα_s_) and in efficacy in Gα_i_, and it showed no response in β-arrestin when compared with PTH1R cells alone (Figure 6; Table 5).

**TABLE 5:** Consequences of RAMP2 and RAMP3 in the potency and efficacy of PTH (1-84) in different functional assays (Figure 6).

Cell line	pEC50 ± SEM			
	cAMP (Gαs)	ß-arrestin	Calcium influx (Gαq)	cAMP + PTX
PTH1R Parental	8.20 ± 0.11	8.40 ± 0.12	ND	7.80 ± 0.11
PTH1R + RAMP2	9.24 ± 0.08***	8.10 ± 0.11	7.38 ± 0.15*	9.01 ± 0.10***
PTH1R + RAMP3	7.55 ± 0.13***	ND	ND	7.51 ± 0.13

Cell line	Efficacy (%) ± SEM			
	cAMP (Gαs)	ß-arrestin	Calcium influx (Gαq)	cAMP + PTX
PTH1R Parental	86.6 ± 4.40	6.08 ± 0.26	ND	85.5 ± 4.17
PTH1R + RAMP2	95.5 ± 2.77	103.5 ± 4.77***	82.2 ± 5.70*	94.3 ± 3.60
PTH1R + RAMP3	72.2 ± 4.83*	ND	ND	71.5 ± 4.41**

ND: could not be determined.

Data were analyzed using comparison of fits (GraphPad Prism) for non-linear regression curves and three-parameter logistic curve (*P < 0.05, **P < 0.01, and ***P < 0.001).

### PTH (1-17)

**FIGURE 7:**
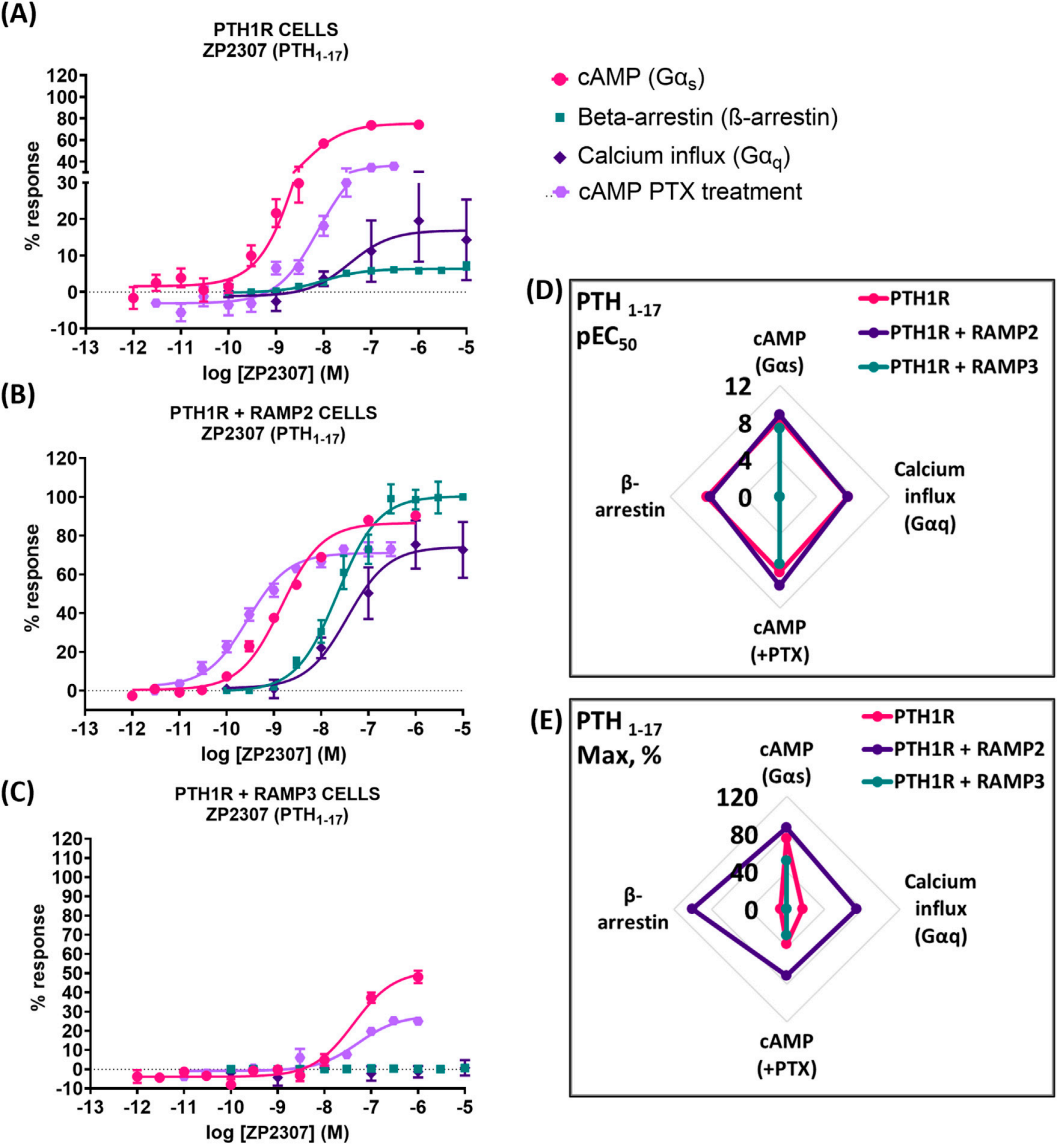
Consequences of RAMP2 and RAMP3 in the potency and efficacy of PTH (1-17)/ZP2307 in different functional assays. Dose–response curves of PTH (1-17) in different second messenger pathways: in CHO-K1 cells overexpressing **(A)** PTH1R alone (mock), **(B)** PTH1R with RAMP2, and **(C)** PTH1R with RAMP3. Spider diagrams of **(D)** the potency and **(E)** the efficacy of PTH (1-17) in those assays, as extracted from the dose–response curves. Data are derived from curves constructed from at least 3–4 independently replicated studies, each consisting of two replicate measurements at each of the 11 ligand concentrations. Data were analyzed using comparison of fits (GraphPad Prism) for non-linear regression curves and three-parameter logistic curve (*p < 0.05, **p < 0.01 ***p < 0.001). All curves were expressed as a % of the positive controls/maximal response. Controls: cAMP studies: forskolin (100 μM), calcium influx studies: ATP (100 μM), β-arrestin-2 recruitment studies: maximal response at the highest dose (1 μM).

Very similar effects were observed when we used a shorter, chemically modified cyclic analog of PTH (1-34) and PTH (1-17), also known as ZP2307 (Neerup et al., 2011). PTH1R RAMP2 cells showed a significant increase in both potency and efficacy in cAMP accumulation (Gα_s_) and Gα_i_, whereas PTH1R RAMP3 cells showed a significant decrease in both cAMP accumulation (Gα_s_) and only in the efficacy of ZP2307 in Gα_i_ (Figure 7; Table 6). In β-arrestin, like all the other peptides, ZP2307 had a significantly increased efficacy in PTH1R RAMP2 cells when compared to PTH1R alone, whereas PTH1R RAMP3 showed no response (Figure 7; Table 6). Finally, similar to PTH (1-34), a significant increase in efficacy but not in potency was observed in calcium influx when PTH1R RAMP2 cells were stimulated with ZP2307 compared to PTH1R alone. PTH1R RAMP3 cells showed no response (Figure 7; Table 6).

**TABLE 6:** Consequences of RAMP2 and RAMP3 in the potency and efficacy of PTH (1-17)/ZP2307 in different functional assays (Figure 7).

Cell line	pEC50 ± SEM			
	cAMP (Gαs)	ß-arrestin	Calcium influx (Gαq)	cAMP + PTX
PTH1R Parental	8.40 ± 0.06	8.00 ± 0.15	7.45 ± 0.86	8.11 ± 0.10
PTH1R + RAMP2	8.84 ± 0.04***	7.65 ± 0.08	7.45 ± 0.29	9.56 ± 0.07***
PTH1R + RAMP3	7.38 ± 0.11***	ND	ND	7.28 ± 0.14

Cell line	Efficacy (%) ± SEM			
	cAMP (Gαs)	ß-arrestin	Calcium influx (Gαq)	cAMP + PTX
PTH1R Parental	75.4 ± 2.23	6.36 ± 0.30	16.9 ± 5.19	37.2 ± 1.98
PTH1R + RAMP2	86.6 ± 1.49***	100.5 ± 2.81***	74.2 ± 7.11**	71.1 ± 1.38***
PTH1R + RAMP3	51.4 ± 2.94***	ND	ND	28.1 ± 2.25**

ND: could not be determined.

Data were analyzed using comparison of fits (GraphPad Prism) for non-linear regression curves and three-parameter logistic curve (*P < 0.05, **P < 0.01, and ***P < 0.001).

### PTHrP (1-108) and PTHrP (1-141)

**FIGURE 8:**
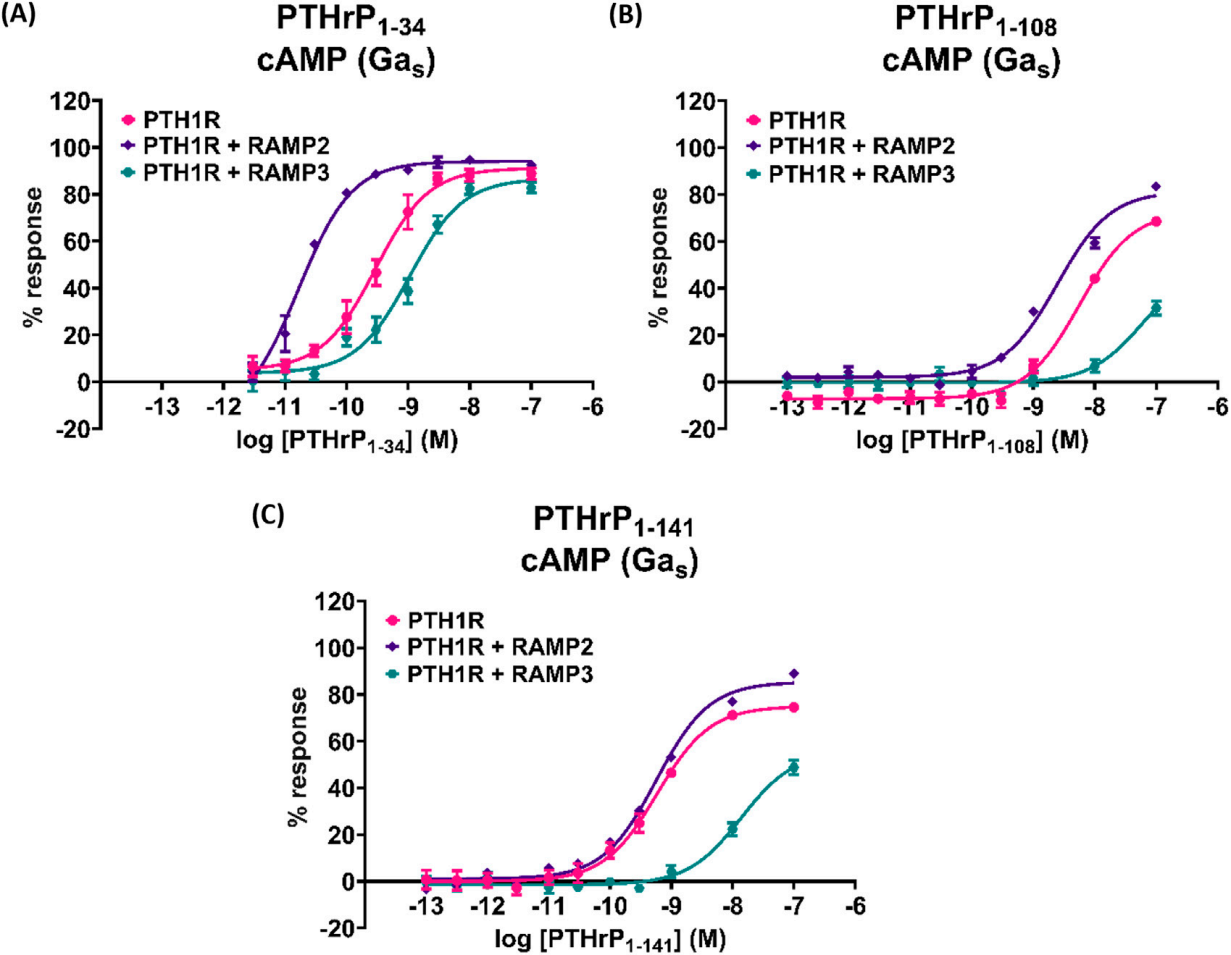
Effects of larger PTHrP analogs including the PTHrP (1-108) and the full-length PTHrP (1-141) in cAMP accumulation studies compared to the more widely studied PTHrP (1-34). Dose–response curves of **(A)** PTHrP (1-34), **(B)** PTHrP (1-108), and **(C)** PTHrP (1-141) in cAMP accumulation studies, in CHO-K1 cells overexpressing PTH1R alone (mock), PTH1R with RAMP2, and PTH1R with RAMP3. Data are derived from curves constructed from at least 3–4 independently replicated studies, each consisting of two replicate measurements at each of the 11 ligand concentrations. Data were analyzed using comparison of fits (GraphPad Prism) for non-linear regression curves and three-parameter logistic curve. All curves were expressed as a % of the positive control (forskolin (100 μM)). *p < 0.05, **p < 0.01, and ***p < 0.001, for comparisons against PTHrP (1-34); ^$^ *p* < 0.05, ^$$^ *p* < 0.01, and ^$$$^ *p* < 0.001, for comparisons against PTH1R cells alone.

We also tested the effects of larger PTHrP analogs including PTHrP (1-108) and full-length PTHrP (1-141). Compared to the more widely studied PTHrP (1-34), these have statistically significant decreased potency and efficacy in activating PTH1R alone in cAMP accumulation studies (Figure 8; Table 7). The presence of RAMP2 significantly increased the potency and efficacy of PTHrP (1-108) and the efficacy but not the potency of PTHrP (1-141) compared with their activity on PTH1R alone. However, here we show a statistically significant reduction in both potency and efficacy of cAMP activation in the presence of RAMP3 compared to the stimulation of PTH1R alone by PTHrP (1-108 and 1-141). Due to the limited availability of these peptides, we were unable to study the effects on other signaling pathways (calcium and β-arrestin).

**TABLE 7:** Effects of larger PTHrP analogs including the PTHrP (1-108) and the full-length PTHrP (1-141) in cAMP accumulation studies compared to the more widely studied PTHrP (1-34) (Figure 8).

Cell line	pEC50 ± SEM		
	PTHrP 1–34	PTHrP 1–108	PTHrP 1–141
PTH1R Parental	9.53 ± 0.08	8.25 ± 0.07***	9.23 ± 0.06*
PTH1R + RAMP2	10.8 ± 0.07	8.61 ± 0.06***,$$$	9.25 ± 0.04***
PTH1R + RAMP3	8.98 ± 0.09	Ambiguous fit	7.86 ± 0.10***,$$$.

Cell line	Efficacy (%) ± SEM		
	PTHrP 1–34	PTHrP 1–108	PTHrP 1–141
PTH1R Parental	91.1 ± 2.86	72.8 ± 2.85***	74.9 ± 2.42***
PTH1R + RAMP2	94.0 ± 1.51	81.4 ± 2.03***,$	85.3 ± 1.45***,$$$
PTH1R + RAMP3	86.7 ± 3.34	Ambiguous fit	55.7 ± 3.59***,$$$

Data were analyzed using comparison of fits (GraphPad Prism) for non-linear regression curves and three-parameter logistic curve.

*p < 0.05, **p < 0.01, and ***p < 0.001 for comparisons against PTHrP (1-34); $*p* < 0.05, $$*p* < 0.01, and $$$*p* < 0.001 for comparisons against PTH1R cells alone.

## Discussion

In this study, we investigated the effects of receptor activity-modifying proteins (RAMPs) on the pharmacology of the parathyroid hormone 1 receptor (PTH1R) using a variety of PTH/PTHrP-related ligands. Our results demonstrate that RAMP2 and RAMP3 differentially interact with PTH1R and modulate its responses to these ligands. RAMP2 enhances β-arrestin recruitment and calcium signaling, whereas RAMP3 inhibits these pathways and appears to retain PTH1R intracellularly. We also show that the presence of RAMP2 differentially modulates the potency and efficacy of PTH/PTHrP-related peptides in activating G-proteins and recruiting β-arrestins. Furthermore, we report the effects of full-length PTHrP ligands, PTHrP (1–108), and PTHrP (1-141), on PTH1R signaling, and the influence of RAMPs on these responses. Our findings highlight the complex role of RAMPs in modulating PTH1R function and suggest that targeting PTH1R/RAMP2 maybe of potential therapeutic value.

Biased agonism is well studied on PTH1R, with differing activation and duration in cAMP, calcium, and β-arrestins resulting in distinct physiological outcomes (Luttrell et al., 2018). The goal of translating biased agonist ligands into viable therapeutics is to increase bone mass while reducing calciotropic effects in conditions such as osteoporosis. However, how accessory proteins, such as the RAMP family, play a role in biased agonism of PTH1R is less well studied. PTH1R and RAMP interactions have previously been identified (Lorenzen et al., 2019; Harris et al., 2021). Nemec et al. (2022) undertook the first comprehensive pharmacological study of PTH1R and RAMP2 using PTH (1-34) and PTHrP (1-34). In the present study, we investigated and expanded upon the possible effects of RAMPs on PTH1R pharmacology with a larger repertoire of PTH-derived ligands.

Our data show that both RAMP2 and RAMP3 form an interaction with PTH1R, but not RAMP1, which is consistent with observations by Harris et al. (2021). Our FRET data confirm the preference of PTH1R for interacting with RAMP2 and less so with RAMP3, whereas no interaction was shown with RAMP1 (Figures 2A, B), as recently reported by Nemec et al. (2022). We also observed different stoichiometric ratios of RAMP2 and RAMP3 interactions with PTH1R (Table 1), which were similar to changes in GPCR/RAMP stoichiometry previously observed in the class-C GPCR calcium-sensing receptor (Desai et al., 2014). The rationale for a FRET-based stoichiometric approach, as opposed to a pure FRET efficiency method, is that the latter is expressed in arbitrary units and cannot determine whether a low FRET signal is due to the absence of interaction between the components or to a local excess of donor and acceptor molecules. Using FRET stoichiometry, we can estimate the fraction of acceptor molecules in a complex with donor molecules and the fraction of donor molecules in a complex by measuring the donor fluorescence lost due to energy transfer (Hoppe et al., 2002). This eliminates the need for acceptor photobleaching to determine total donor concentrations and allows for repeated measurements from the same cells (Hoppe et al., 2002).

RAMP2 interaction with PTH1R does not alter cell surface expression of PTH1R, confirming the observations by Christopoulos et al. (2003). However, RAMP3 appears to almost completely ablate cell surface expression of PTH1R (Figure 2D) but not the whole-cell PTH1R expression (Figure 2C), possibly implying a retention preventing forward trafficking of PTH1R to the cell surface or an increase in PTH1R internalization/recycling, as recently shown by Mackie et al. (2019). To confirm these, further experimental studies are required, such as real-time microscopy. It has previously been reported that PTH1R localizes in sub-cellular compartments including the nucleus (Pickard et al., 2007). In recent years, it has been increasingly recognized that GPCR signaling can continue after endocytosis. This phenomenon, called endosomal signaling, challenges the traditional view that GPCR signaling is mainly confined to the cell surface. The effects of the subcellular localization of PTH1R have previously been shown to affect ligand binding (Peña et al., 2022; Ferrandon et al., 2009). Studies have shown that PTHrP(1–36) or PTHrP (1-34) analog (abaloparatide) primarily exerted its effects on the cell surface, whereas PTH(1-34) was more prone to endosomal internalization, resulting in an extended elevation of cAMP levels in cells overexpressing PTH1R (Ferrandon et al., 2009; Hattersley et al., 2016).

Using antibody-capture SPA in COS-7, we showed that PTH1R with RAMP2 induces different responses to PTH (1-34) and PTHrP (1-34) compared to PTH1R alone (Figure 3), illustrating an example of ligand-induced functional selectivity responses for a receptor/RAMP complex, as observed previously (Weston et al., 2016; Weston et al., 2015). RAMP2 increased PTH-stimulated maximal activation of Gα_s_ and Gα_i_ without changing Gα_q_. PTHrP induced a different pattern of changes, where PTHrP-stimulated Gα_s_ efficacy increased significantly without changes in Gα_i_, Gα_q_, or potency. PTH induced greater maximal activation of Gα_s_ and Gα_i_ than PTHrP and slight Gα_q_ activation that was absent with PTHrP. As PTH and PTHrP are known to bind to the same PTH1R but induce different tissue/organism effects (Schluter, 1999; Clemens et al., 2001), this provides quantitative confirmation of ligand-induced functional selectivity at the level of G-protein activation by different physiological ligands binding to the same receptor (Schwindinger et al., 1998). COS-7 cell model was used as an aspect of the study due to its ability to express high levels of receptor at the cell surface and express a full complement of G-protein in the cell (Weston et al., 2015). COS-7 may not be the optimal cell system to perform studies on PTH1R as receptor recycling and desensitization (Sneddon et al., 2004) have not been observed in these cells; however, in this experiment, we studied the activation of the G-proteins in isolated membrane fragments, where internalization and desensitization are not studied. To address these issues, the whole functional assay CHO-K1 cells were used, which have previously been used to study internalization and desensitization (Wang et al., 2007; Romero et al., 2010).

Nemec et al. showed that RAMP2 specifically and selectively enhanced the activation kinetics of Gα_s_ and Gα_i3_ proteins by PTH (1-34) (Nemec et al., 2022), which is consistent with our data (Figure 3), showing larger maximal responses by PTH (1-34) on PTH1R Gα_s_ activation and Gα_i_ sensitivity in the presence of RAMP2. We also show a significant reduction in potency with no change in efficacy of Gα_q_ activation by RAMP2 (Figure 3), whereas others did not observe any differences. For PTHrP (1-34), we observed an increase in efficacy of only Gα_s_ with no alterations in Gα_i_ signaling (Figure 3), in contrast to observations where RAMP2 did not alter PTHrP (1-34) G-protein activation by PTH1R. These discrepancies could reflect the different detection methods used.

The kinetics of cAMP activation play a crucial role in the differential effects of PTH and PTHrP. PTH (1-34) has been shown to induce prolonged cAMP activation compared to PTHrP (1-34) (Ferrandon et al., 2009; Dean et al., 2008). This prolonged cAMP activation is associated with the bone anabolic effects of PTH (1-34). In the presence of RAMP2, our results show that PTH (1-34) has higher potency and efficacy than PTH1R alone, suggesting an even longer duration/magnitude of cAMP activation. However, PTHrP (1-34) exhibits increased potency but unchanged efficacy in the presence of RAMP2, indicating a different pattern of cAMP activation kinetics, which could possibly be as a result of altered receptor internalization and endosomal signaling (Ferrandon et al., 2009). A limitation of our study is that we did not directly examine the kinetics of cAMP activation, which could be addressed in future studies to further elucidate the role of RAMPs in modulating the temporal aspects of PTH1R signaling.

The presence of RAMP2 significantly enhances the maximal response of β-arrestin recruitment by PTH1R for all ligands tested (Figures 6–8, 9), which is consistent with the previous work by Nemec et al. (2022). Our data suggest that RAMP2 has a universal effect on augmenting β-arrestin recruitment and PTH1R desensitization. Increased β-arrestin activity has been correlated with bone anabolic effects (Gesty-Palmer et al., 2006; Gesty-Palmer et al., 2009), and the β-arrestin selective agonist D-Trp12,Tyr34-bPTH (7-34) has been shown to increase bone formation without activating G-proteins, inducing hypercalcemia, or increasing the markers of bone resorption (Bohinc and Gesty-Palmer, 2011). These findings highlight the potential role of β-arrestin signaling in the regulation of bone metabolism and suggest that targeting β-arrestin recruitment through the PTH1R/RAMP2 complex may be a promising strategy for the development of novel bone anabolic therapies.

Our results also demonstrate that PTH (1-34) induces changes in the efficacy and/or potency of all downstream second messenger used (cAMP, calcium, and β-arrestin), whereas PTHrP (1-34) elicits a dramatic increase in β-arrestin recruitment but no change in other second messengers. Interestingly, PTHrP (1-34) has been shown to have comparable bone anabolic effects compared to PTH (1-34) (Martin, 2016; Cosman et al., 2022). Our data may suggest that the bone anabolic effects of PTH (1-34) and PTHrP (1-34) could be mediated through the PTH1R/RAMP2 complex rather than PTH1R alone, with similar effects observed for PTH (1-84). Additionally, the differential effects of PTH (1-34) and PTHrP (1-34) on G-protein and β-arrestin signaling in the presence of RAMP2 could provide new insights into the mechanisms underlying their distinct bone anabolic properties. Our findings suggest that the PTH1R/RAMP2 complex may be a key mediator of the bone anabolic effects of PTHrP (1-34) and potentially other PTH1R ligands. Further research is needed to elucidate the precise role of RAMP2 in regulating PTH1R signaling and its implications for bone physiology and disease.

The effects of RAMP3 on PTH1R have not been reported previously. As described above, we see an interaction between PTH1R and RAMP3. The cell-surface ELISA data show reduced PTH1R expression, which may suggest a receptor retention effect by RAMP3, yet we see responses by PTH1R/RAMP3, albeit reduced, compared to PTH1R/RAMP2. β-Arrestin and calcium signaling are absent in the presence of RAMP3 compared to PTH1R/RAMP2 and PTH1R alone. In the presence of RAMP3, the lack of cell surface trafficking and second messenger signaling is inconsistent. However, there have been reports of intracellular PTH1R activation (Vilardaga et al., 2023). Nonetheless, RAMP3 has also been reported to be an early response gene to PTH stimulation, further suggesting a potential important regulatory function of the PTH1R/RAMP3 interaction (Phelps et al., 2005).

During this study, we were also able to test less commonly explored PTHrP (1–108) (full-length analog) and PTHrP (1-141) (full-length) peptides (Hammonds et al., 1989). These full-length PTHrP ligands have not been widely studied due to challenges in their synthesis. Compared to the more widely studied PTHrP (1-34), these have lower potency and efficacy in activating PTH1R in cAMP accumulation studies. The presence of RAMP2 increased their potency and/or efficacy when compared to PTH1R alone. However, consistent with our previous observations, RAMP3 reduces the response of PTH1R to these ligands when compared to PTH1R alone. Due to the limited availability of these peptides, we were unable to study the effects on other signaling pathways (calcium and β-arrestin). This strengthens the implication that RAMP2 plays a broad role in modulating PTH1R pharmacology across a variety of ligands.

The study by Kadmiel et al. provides valuable *in vivo* evidence supporting the physiological relevance of RAMP2-GPCR interactions beyond the canonical AM-CLR signaling paradigm (Kadmiel et al., 2017). The reduced PTH1R expression in Ramp2−/− placentas and the blunted response to very large doses (500 μg/kg) of systemic PTH administration in Ramp2 +/− adult females complement our *in vitro* data demonstrating that RAMP2 modulates PTH1R signaling (Kadmiel et al., 2017). These data suggest how these *in vitro* changes in ligand bias reported here may influence *in vivo* functions; however, this needs to be explored in more detail using physiologically relevant levels of PTH and/or PTHrP.

In summary, our findings highlight the complex role of RAMPs in modulating PTH1R signaling and function. We show that RAMP2 and RAMP3 differentially interact with PTH1R and modulate its responses to a diverse range of PTH/PTHrP-related ligands. The presence of RAMP2 enhances β-arrestin recruitment and calcium signaling, whereas RAMP3 appears to reduce cell surface expression of PTH1R, and subsequently, reduced PTH1R signaling is observed. The differential effects of PTH (1-34) and PTHrP (1-34) on G-protein and β-arrestin signaling in the presence of RAMP2 could provide new insights into the mechanisms underlying their distinct bone anabolic properties. Moreover, our data also warrant detailed understanding of whether the kinetics of cAMP activation is differentially modulated by RAMP2 for PTH (1-34) and PTHrP (1-34) and can likely contribute to their divergent physiological effects. One constraint in our research is understanding if the binding affinities of these ligands are altered via allosteric modulation by RAMPs, especially RAMP2, and whether it may impact the interpretation of the signaling data. Overall, our data suggest that targeting the PTH1R/RAMP2 complex may be a promising strategy for the development of novel bone anabolic therapies by potentially leveraging functional selectivity. Further research using functional readouts in primary cells, and appropriate animal models, including knockout mice, will be crucial to elucidate the physiological relevance of these findings and their potential therapeutic implications.

## Materials and methods

### Materials

Reagents were purchased from the respective manufacturers: Ham’s F12-K (Kaighn’s) medium, RPMI medium, sodium pyruvate, penicillin/streptomycin, fetal bovine serum (FBS), Opti-MEM™ (Reduced Serum Medium), Lipofectamine 3000 (GIBCO-Invitrogen-Life Technologies, Carlsbad, CA), and AssayComplete™ Cell Culture Kit-107 (DiscoverX, California, United States); ATP, forskolin, IBMX, and Pertussis toxin (Sigma Aldrich, St. Louis, MO); G418 (Thermo Fisher Scientific, Loughborough, United Kingdom); and rabbit anti-goat-HRP antibody and goat anti-mouse-HRP antibody (Dako, Denmark); LANCE cAMP Detection kit (Perkin Elmer, Massachusetts, United StatesS), FLIPR Calcium 6 Evaluation Kit (Molecular Probes, Oregon, United States), and PathHunter® Detection Kit (DiscoverX, California, United States). CHO-K1 and COS-7 cell lines (ATCC, Virginia, United States) and PathHunter CHO-K1 PTH1R β-arrestin cell line (DiscoverX, California, United States); PTH (1-34), PTH (1-84), and PTHrP (1-34) (Bachem Holding, Bubendorf, Switzerland). PTH (1-17), also known as ZP2307, was a gift from Dr Rasmus Just of Zealand Pharma. Truncated PTHrP peptides were those developed and produced by Professor Jack Martin (Hammonds et al., 1989).

### Cell transfections and cell line generation

COS-7 cells were grown to confluency in DMEM with GlutaMAX™, supplemented with 10% FCS and 1x penicillin/streptomycin in a 5% CO_2_ incubator at 37°C. The cells were harvested using trypsin/EDTA (Sigma), washed with PBS, and re-suspended in electroporation buffer (composition [mM] 20 HEPES, 135 KCl, 2 MgCl_2_, 2 ATP, 5 glutathione, and 0.5% Ficoll 400 adjusted to pH 7.6 using KOH) at a concentration of ∼4 million cells into 4-mm gap electroporation cuvettes (York Biosciences, United Kingdom), and the required DNA was added (5 μg PTH1R, 15 μg RAMP constructs). The cells were then electroporated at 0.25 kV and 960 µF using a Gene Pulser (Bio-Rad) and cultured for 48 h.

CHO-K1 PTH1R β-arrestin cells were grown in AssayComplete™ Cell Culture Kit-107, containing all necessary supplements and antibiotics, at 37°C in 5% CO_2_, and were sub-cultured in a 1:10 ratio every 3–4 days. To be used in the functional assays (cAMP accumulation, β-arrestin recruitment, and calcium mobilization), cells were transfected with C-tagged cerulean RAMP (RAMP1-3) constructs or mock vector using Lipofectamine 3000 following the manufacturer’s guidelines. Cells were selected using 0.5 mg/mL G418 48 h after transfection and cultured in the aforementioned growth media for 1 week. RAMP-expressing cells were validated using fluorescence imaging using the EVOS microscope and population enrichment by fluorescence-assisted cell sorting using the FACS Aria II (BD Biosciences, New Jersey, United States) (Supplementary Methods, Supplementary Figure S1). This was done twice (two separate sorts, Supplementary Figure S2). Mock transfected PTH1R parental cells were used as the negative control, for the gating of the positive population of cells, and in all functional assays.

### Membrane preparations

Cell membrane extractions were performed using COS-7 cells transfected with PTH1R and different RAMPs (described above) and used in Scintillation proximity assay. At 48 h post transfection, the cells were homogenized using a Dounce homogenizer using ice cold PBS and centrifuged at 300 *g* for 10 min at 4°C in a final volume of 40 mL. The supernatant was collected in a fresh tube and centrifuged at 50,000 g for 25 min at 4°C. The resulting pellet was re-suspended in ice cold SPA buffer (50 mM HEPES, 100 mM NaCl, 5 mM MgCl2, 0.5% BSA, and pH 7.4). Total protein concentrations were measured using the bicinchoninic acid assay (Sigma).

### Preparation of constructs for FRET and COS-7 cell transfection

FRET studies were performed with minor modifications to previously published methods (Desai et al., 2014). Citrine or Cerulean cDNAs were engineered into a pcDNA3.1 vector (Invitrogen) between the Not1 and Xho1 restriction enzyme sites. RAMPs and PTH1R were engineered into pcDNA3.1 Cerulean and Citrine vectors, respectively, excluding their stop codons between the Kpn1 and Not1 and HindIII and Not1 restriction enzyme sites so that the fluorophores were present at the C-terminal of RAMP/PTH1R. As a negative control, pcDNA 3.1 containing Citrine alone were co-transfected with a pcDNA3.1 RAMP Cerulean vector. As a positive control, we created a pcDNA3.1 vector containing a Cerulean cDNA fusion construct followed by 18 amino acid linker sequence and then Citrine cDNA.

COS-7 cells were grown to confluency and harvested using trypsin/EDTA (Sigma), washed with PBS, and re-suspended in electroporation buffer (composition [mM] 20 HEPES, 135 KCl, 2 MgCl2, 2 ATP, 5 glutathione, and 0.5% Ficoll 400 adjusted to pH 7.6 using KOH) at a concentration of ∼3.5–4 million cells into 4-mm gap electroporation cuvettes (York Biosciences, United Kingdom), and the required concentration of DNA was added (10 µg receptor, 15 µg RAMP constructs). The cells were then electroporated at 0.25 kV and 960 µF using a Gene Pulser (Bio-Rad) and cultured for 72 h in 35-mm glass-bottom plates (Ibidi, München), after which they were fixed with 4% PFA and mounted with Mowiol. COS-7 cells were transfected with C-tagged Citrine PTH1R and C-tagged Cerulean RAMP in pcDNA 3.1 vector and grown in 35-mm glass-bottom plates (Ibidi, München) and then fixed and mounted. As a positive control, a fusion of Cerulean–Citrine was created in pcDNA3.1 vector. Cells were excited, and images were captured for analysis.

Images were captured using a Zeiss Plan apo 63×/1.4 oil immersion lens on a Zeiss LSM 510 inverted laser scanning confocal fluorescence microscope fitted with an argon laser at room temperature. Confocal images of the fluorescent proteins were acquired using an argon laser together with an HFT458/514 nm dichroic, a NFT515 nm beam splitter, pin hole set to 496 µm, detector gain 550, and individually as a separate channel under the following conditions: Cerulean was excited using the 458-nm laser line with a 100% laser intensity and a band pass BP480–520 emission filter, Citrine was excited using the 514-nm laser line attenuated to 20% laser intensity and a band pass BP535–590 emission filter, and FRET was excited using the 458-nm laser line with a 100% laser intensity and a BP480–520 emission filter. All fluorescence channels were scanned and collected line-by-line with a mean of 1.

Cerulean and Citrine fluorescence bleed-through into the FRET channel were calculated using FRET and co-localization analyzer plugin for ImageJ (Feige et al., 2005). NFRET calculations for FRET efficiency for sensitized emission were carried out using pixel-by-pixel analysis by PixFRET plugin for ImageJ (Cussac et al., 2004). The threshold for pixel intensity to be included in analysis was set to 1.5 times the background intensity.

The following equation was used to calculate the FRET efficiency:

$$\text{NFRET}=\frac{\text{FRET}-\left[\text{CFP}\times \left({\text{CFP}}_{\text{BT}}\right)\right]-\left[\text{YFP}\times \left({\text{YFP}}_{\text{BT}}\right)\right]}{\sqrt{\left(\text{CFP}\times \text{YFP}\right)}}.$$

BT=bleed through.

The correction factors calculated were as follows: β (proportionality constant relating donor fluorescence detected at the acceptor emission relative to that detected at the donor emission): 0.31, α (proportionality constant relating acceptor fluorescence at the acceptor excitation to the donor excitation): 0.126, γ (ratio of the extinction coefficient of the acceptor to the donor at the donor excitation): 0.3, and ξ (proportionality constant relating the sensitized acceptor emission to the decrease in donor fluorescence due to FRET): 0.2.

Cell surface FRET was separated from whole-cell FRET by constructing a series of 50-pixel diameter dots around the cell surface of the raw acceptor image using the selection tool of ImageJ. Each dot was taken as an ROI, and the combined ROIs for each image were used to calculate mean membrane NFRET and stoichiometry values. All FRET-based stoichiometric analyses were performed as previously described (Hoppe et al., 2002) using ImageJ software.

### Whole cell and cell surface expression ELISA

Cells (CHO-K1 parental or stable cells generated as described in Cell Line Generation section above) were seeded at 150,000 cells/well into 24-well plates coated with poly-D-lysine. Following 48 h growth in complete growth media, media was replaced with 4% formaldehyde for 15 min to fix the cells. Cells were washed three times with 500 µL phosphate-buffered saline (PBS) and incubated with 1% BSA in PBS for 45 min to prevent nonspecific antibody binding. To determine receptor expression, 250 µL of primary antibody (mouse anti-PTH1R (ab104832 (1 mg/mL), Abcam, Cambridge, United Kingdom)) at 1:3,000 concentration in 1% BSA PBS was added for 1 h. Cells were washed three times with 500 µL PBS before the addition of the secondary antibody (HRP-conjugated anti-mouse IgG (1.5 mg/mL) (Dako, Denmark)) and diluted 1:2,000 in 1% BSA in PBS for 1 h. Following three further washes with PBS, HRP activity was determined using TMB Substrate Solution (Thermo Fisher Scientific, Massachusetts, United States) according to the manufacturer’s instructions. In the case of whole-cell expression, after fixing, cells were washed three times with 500 µL phosphate-buffered saline (PBS) and permeabilized with 0.1% Triton X-100 in PBS for 30 min at room temperature. Cells were washed three times with 500 µL phosphate-buffered saline (PBS), and the same procedure as the cell surface ELISA was then used.

### Scintillation proximity assay for G-protein activation

Receptor/G-protein activation profiles were determined using a scintillation proximity assay, as described previously (Lan et al., 2009). Briefly, COS-7 cells were transfected with different native untagged human receptors alone or in combination with native untagged human RAMPs1-3 in pcDNA3.1. Receptor concentrations were determined by radioligand binding studies and Western blotting (not shown). For the SPA assay, membranes were incubated with different concentrations of ligands with [35^S^] GTPγ^S^. Polyclonal antibodies to different G-proteins were added (Santa Cruz). Scintillation proximity assay beads coated with secondary antibody were added and measured in a scintillation counter. Dose–response curves were generated from no fewer than two independent replicates at eight different ligand concentrations in three independent studies for each receptor and RAMP comparison.

### Intracellular calcium mobilization assay

To assess the consequences of RAMP expression in calcium influx, calcium mobilization assays were performed in 96-well black, clear-bottom plates (Corning, United States). Forty-eight hours before the assay was performed, CHO-K1 PTH1R β-arrestin cells stably expressing the different RAMPs (generated as explained above) were seeded at 10,000 cells/well in the standard growth medium to give 80% confluency at the time of performing the assay. The medium was replaced with 1% FBS medium 24 h prior to stimulation. After thawing and equilibrating the 10x Calcium 6 assay reagent to RT, it was dissolved in 10 mL (1:10) of loading buffer (1x HBSS buffer, 20 mM HEPES, 10 mM CaCl_2_, and pH adjusted to 7.4). Probenecid was added to the loading dye to give final in-well concentration of 2.5 mM (this prevents the release of the dye from the cells back into to the medium). A total of 100 μL of 1x calcium-6 loading dye was added to all wells and incubated for 2 h at 37°C and 5% CO_2_. All peptide ligands used were diluted in 1x loading buffer. Following incubation, the plate was then transferred directly to the FlexStation3 assay plate reader (Molecular Devices, California, United States) and was allowed to equilibrate at 37°C for 10 min. Traces were collected for 300 s, including a 50-s baseline read prior to peptide addition. All intra-experimental traces were collected in duplicate. The fluorescence values after exposure were subtracted by the basal fluorescence value before exposure, and the data were normalized using ATP (1μΜ)-stimulated controls as 100% response. Dose–response curves were analyzed using a three-parameter logistic curve to determine the EC_50_ values (GraphPad Prism 9 and 10).

### Time-resolved fluorescence resonance energy transfer (TR-FRET) cAMP accumulation

To assess the consequences of RAMP expression in cAMP accumulation, the total cAMP was measured using the TR-FRET LANCE cAMP detection kit (PerkinElmer, AD0264), according to the manufacturer’s directions. The assay was performed using CHO-K1 PTH1R β-arrestin cells stably expressing the different RAMPs (generated as explained above). Aliquots of frozen cells (2 × 10^6^ each) were thawed and prepared in warm stimulation buffer (1 × HBSS, 5 mM HEPES, 0.5 mM IBMX, and 0.1% BSA). Alexa Fluor cAMP specific antibody (1:100 concentration) was added to the cell suspension, and cells were plated (2,500 cells, 6 μL) in a 384-well white opaque microtiter plate (OptiPlates, Perkin Elmer, 6007299). Cells were incubated with serial dilutions (3 μL) of the peptide ligands (agonist) for 30 min at RT. Subsequently, 12 µL detection mix (Europium-Chelate streptavidin/biotinylated cAMP) was added to stop the reaction and induce cell lysis. TR-FRET was detected after an hour of incubation by an EnSight multimode plate reader (Perkin Elmer) at 320/340 nm excitation and 615/665 nm emission. Data were normalized to a forskolin (100 μM)-only control as 100% cAMP accumulation. Dose–response curves were analyzed using a three-parameter logistic curve to determine EC_50_ values (GraphPad Prism 9 and 10).

### Pertussis toxin treatment

For investigation of Gα_i_ modulation, cells were pre-treated with growth media supplemented with 200 ng/μL pertussis toxin (PTX) (Sigma-Aldrich, United States), as was previously shown (Lan et al., 2009; Morfis et al., 2008; Wall et al., 2022). Following an overnight incubation with PTX, cells were frozen down and were used following the cAMP detection procedure above.

### Beta (β)-arrestin recruitment

To assess the consequences of RAMP expression in the recruitment of β-arrestin (β-arrestin-2 isoform), the PathHunter^®^ Detection Kit was used. The assay was performed following the manufacturer’s instruction and by using CHO-K1 PTH1R β-arrestin cells stably expressing the different RAMPs (generated as explained above). More specifically, 20 μL (5,000 cells/well) was added in a 384-well white opaque microtiter plate. Cells were then incubated with serial dilutions (5 μL) of the peptide ligands (agonist) prepared in 1x HBSS +20 mM HEPES buffer for 90 min at RT. An amount of 12.5 μL working detection solution (mix 19 parts of cell assay buffer, five parts of substrate reagent, and one part of substrate reagent 2) was then added to the wells. Chemiluminescence was detected an hour after using the EnSight multimode plate reader. Data were normalized to the maximal response at the highest ligand dose (1 μM) as 100% and to no ligand as 0% response. Dose–response curves were analyzed using the three-parameter logistic curve to determine EC_50_ values (GraphPad Prism 9 and 10).

## Data Availability

The raw data supporting the conclusions of this article will be made available by the authors, without undue reservation.
