# Supplementary material for: Receptor activity-modifying protein modulation of parathyroid hormone-1 receptor function and signaling
Source: Front Pharmacol. 2024 Sep 23;15:1455231. doi: 10.3389/fphar.2024.1455231 (PMC11456535; doi:10.3389/fphar.2024.1455231)
Supplement: Supplementary file 1 [file DataSheet1.docx]

**Supplementary**

**Supplementary methods**

**Receptor concentration quantification**

Receptor concentration for PTH1R receptor membranes used in the SPA assay, were quantified using radioligand binding studies. Homologous competition experiments were performed using 100fM to 1µM of cold ligand against 50pM PTH_1-34_ hot ligand (Perkin Elmer), incubated with 10µg of membranes and 500µM GTPγ^S^ (Sigma-Aldrich) in a final volume of 300µl containing binding buffer (25mM HEPES, 5mM MgCl2, 1mM CaCl2, 250mM sucrose, 0.5% BSA, pH 7.4). Total binding was determined in absence of the cold ligand. After 1hr the reaction was stopped by rapid filtration through filer paper (Whatman GF/C) previously soaked in 0.5% polyethyleneimine, using a filtration apparatus. Filters were rinsed three times with ice cold PBS containing 0.1% BSA and remaining radioactivity was measure using a gamma counter. Values represent mean and SEM of 3-4 experiments performed in duplicate.

**Flow cytometry cell sorting**

Successful expression of RAMP1- Cerulean, RAMP2- Cerulean and RAMP3- Cerulean was verified by monitoring Cerulean fluorescence levels in cells post-transfection. Transfected populations were maintained in F12-K medium (Kaighn’s Modification of Ham’s F-12 medium), containing 0.5 mg/mL of G418 to ensure efficient selection of successfully transfected cells (Figure S1). Following 1 week of antibiotic treatment, cells were sorted based on their Cerulean fluorescence by FACS using Violet 450/40- A laser (Figure S2). Mock transfected PTH1R parental cells were used as negative control and for the gating of the positive population of cells. This resulted in three distinct populations: mock transfected negative control (PTH1R alone), PTH1R/RAMP1-Cerulean, PTH1R/RAMP2-Cerulean and PTH1R/RAMP3-Cerulean co-expressing CHO-K1 cells. Enriched populations were maintained in complete media containing 0.5 mg/mL G418 to ensure stable expression. Following 1 week of selection, sorted populations underwent a second FACS enrichment and sorted populations were again maintained in complete media containing 0.5 mg/mL G418 (Figure S1 and S2).

**Supplementary results**

|  | pKd | Bmax (pmol/mg) |
| --- | --- | --- |
| PTH1R alone | 8.20 ± 0.16 | 1.78 ± 0.77 |
| PTH1R + RAMP2 | 8.70 ± 0.38 | 1.31 ± 0.63 |

Table S1: Ligand binding affinities (pKd = negative logarithm of ligand concentration) and receptor numbers (Bmax - pmol/mg) for the PTH1R expressed in COS-7 cell membranes as measured by homologous radioligand binding assays in the presence and absence of RAMP2. Values represent mean and SEM of 3-4 experiments, each performed in duplicate. There was no significant difference in pKd for any of the receptors in membranes with or without RAMP2. There was no significant difference in Bmax for PTH1R in membranes with or without RAMP2.


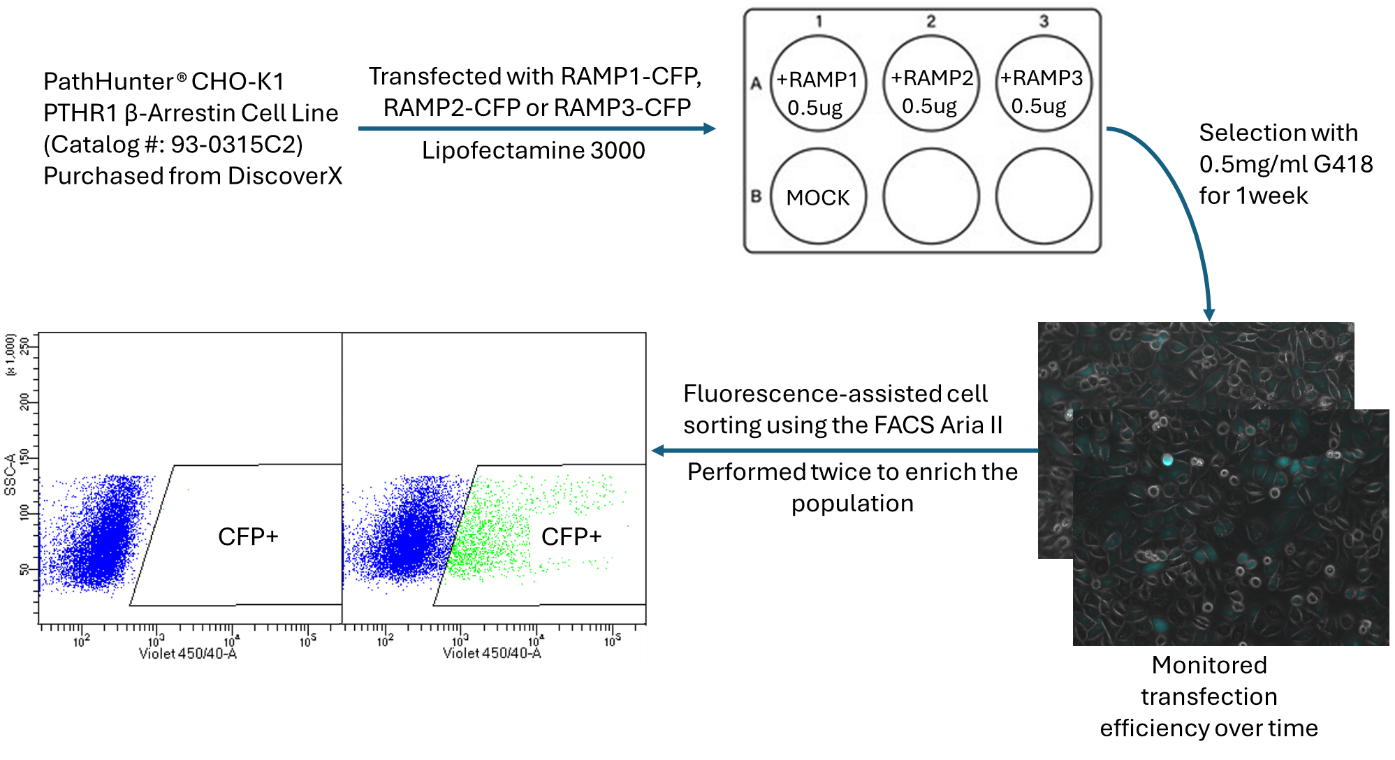


Figure S1: Process of stable cell line generation. CHO-K1 stably expressing PTH1R cells were purchased from DiscoverX. Cells were transfected with C-tagged Ceruleaun RAMPs (RAMP1-3) constructs using Lipofectamine 3000. Cells were selected using 0.5 mg/mL G418 48 hours after transfection and cultured in the aforementioned growth media for 1 week. RAMP expressing cells were validated using fluorescence imaging using the EVOS microscope and population enrichment by fluorescence-assisted cell sorting using the FACS Aria II. This was done twice (two separate sorts).


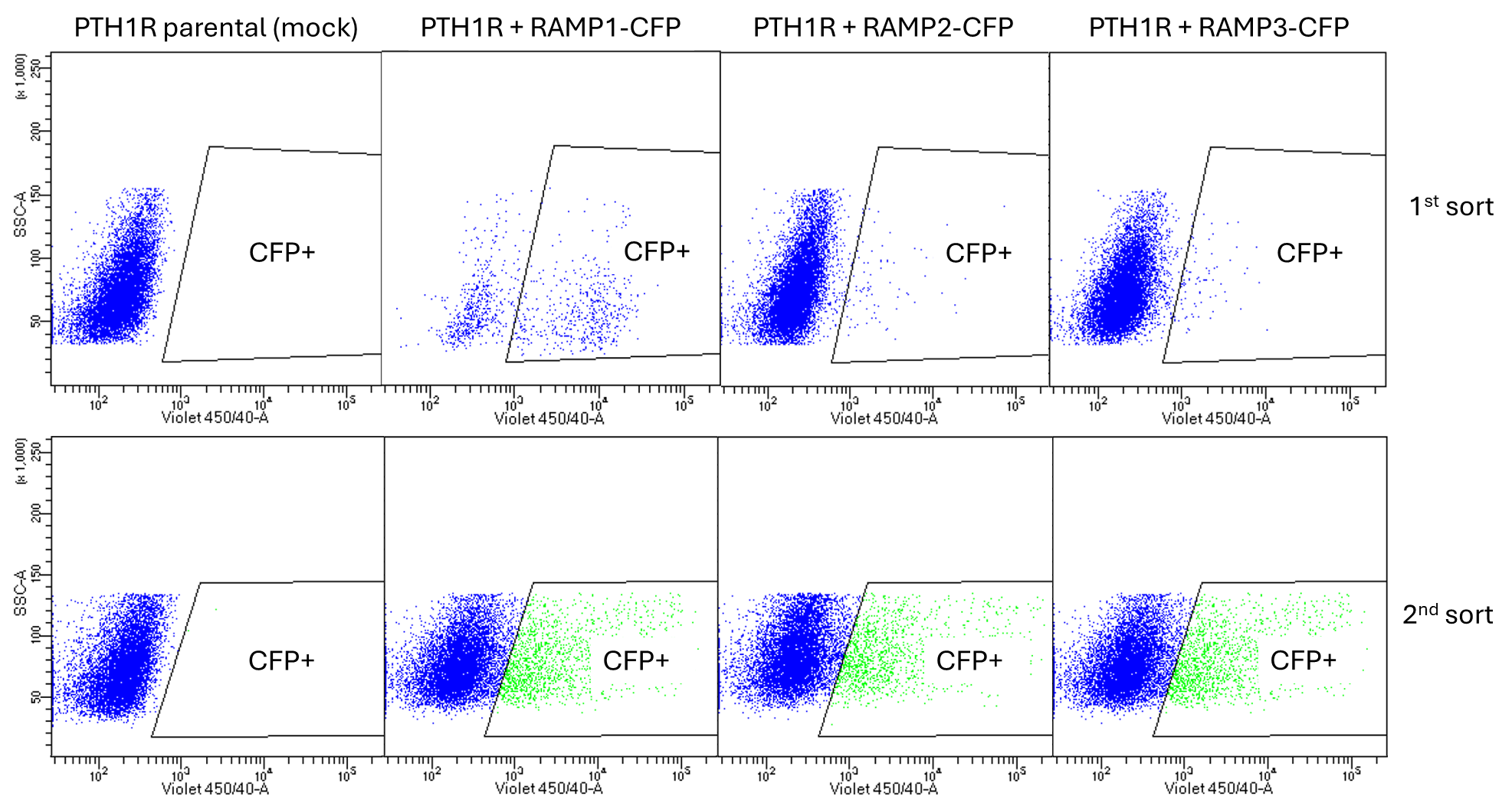


Figure S2: Scatter blots of CFP positive cell sorts. In order to enrich the RAMP positive populations in PTH1R cells transfected with either RAMP1-Cerulean, RAMP2-Cerulean or RAMP3-Cerulean fluorescence-assisted cell sorting using the FACS Aria II was used. Scatter blots using the Violet 450/40 laser, showing the shift of CFP positive cells during the 1^st^ and 2^nd^ cell sort. PTH1R parental (mock transfected) cells were used as negative control and for gating the positive population during the sort.


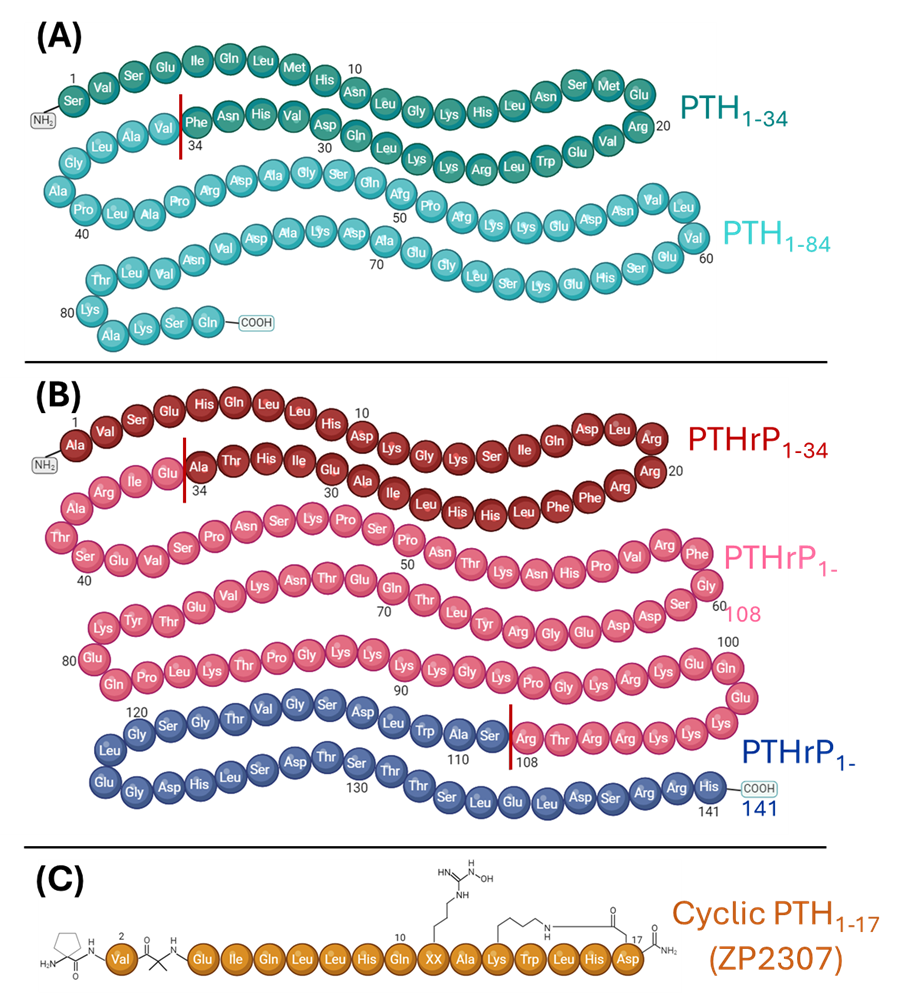


**Figure S3: Primary sequences of PTH and PTHrP analogues. (A)** Amino acid sequence of human PTH_1-34_ and PTH_1-84_. The line separates the PTH_1-34_ amino acid segment from the full-length peptide (PTH_1-84_). **(B)** Amino acid sequence of human PTHrP_1-34_ and PTHrP_1-108_ and PTHrP_1-141_. The lines separate the PTHrP_1-34_  and PTHrP_1-108_ amino acid segments from the full-length peptide PTHrP_1-141_. **(C)** Amino acid sequence of PTH novel human cyclic PTH_1-17_ analogue (ZP2307).
